# Molecular circuit between *Aspergillus nidulans* transcription factors MsnA and VelB to coordinate fungal stress and developmental responses

**DOI:** 10.1371/journal.pgen.1011578

**Published:** 2025-07-17

**Authors:** Emmanouil Bastakis, Jennifer Gerke, Seyma Özkan, Rebekka Harting, Tanja Lienard, Christoph Sasse, Emmanouil S. Xylakis, Merle Aden, Anja Strohdiek, Gabriele Heinrich, Verena Grosse, Gerhard H. Braus

**Affiliations:** Department of Molecular Microbiology and Genetics, Institute for Microbiology and Genetics, University of Göttingen, Göttingen, Germany; University of Georgia, UNITED STATES OF AMERICA

## Abstract

Development and secondary metabolism of the filamentous fungus *Aspergillus nidulans* are tightly controlled by concerted actions of several master regulator transcription factors (TFs). The connection between fungal development and cellular stress response programs is often elusive. Here we show that the zinc finger TF MsnA, which controls salt-stress response, is a novel major regulator of fungal development. A molecular circuit among MsnA and the velvet domain regulator VelB was discovered, which mutually fosters the actions of both regulatory proteins during development. Chromatin immunoprecipitation coupled with next generation sequencing (ChIP-seq) and gene expression studies have revealed that MsnA controls the expression of several genes encoding key transcriptional regulators of asexual as well as sexual development. The double mutant of *msnA* with *velB* showed that both genes share an additive genetic relationship, under normal and salt stress conditions, with each protein to control distinct phenotypical features. In addition, MsnA directly and indirectly affects the synthesis of specific secondary metabolites relevant for fungal defense against other organisms and growth, in addition to salt-stress responses. Moreover, the expression of genes encoding the epigenetic regulators VapA, VipC and LaeA are also directly controlled by MsnA. The VapA-VipC-VapB methyltransferase signal transduction complex promotes asexual differentiation, while the VeA-VelB-LaeA complex balances light response, development and the secondary metabolism of the fungus. MsnA is therefore placed at a novel prominent position of the central regulatory network, which coordinates stress responses with the developmental and metabolic fate of the fungus.

## Author summary

Filamentous fungi, such as *Aspergillus nidulans*, receive several stress stimuli from their environment during development. The coordination between the development and various stress responses is orchestrated by TF proteins, which can directly control the expression of genes. Our work focuses on the MsnA transcriptional regulator, which interconnects growth, secondary metabolism and stress in *A. nidulans*. Our study shows that MsnA directly controls the expression of genes (by its *in vivo* association to their promoter regions) encoding for prominent regulators of development and stress signaling of the fungus. Moreover, MsnA can influence the synthesis of secondary metabolites crucial for the defense and the growth of the fungus via its direct binding to regulatory elements. Lastly, we found that MsnA is a central part of a newly discovered transcriptional hub, where the expression of several prominent regulators of fungal development such as the velvet-domain proteins and known epigenetic methyltransferases such as VipC and LaeA can be orchestrated. Our work highlights the diverse new roles that the MsnA TF has, as a central regulatory protein acting on the stage of development and stress response of the filamentous fungus of *A. nidulans*.

## Introduction

Fungi are sessile organisms, which have to react to various environmental signals to survive and reproduce successfully. Stress signals have to be perceived and mediated by regulatory proteins to change gene expression and induce specific responses. TFs are at the end of these pathways, which control the gene expression of proteins, which are associated with specific stress responses. The filamentous fungus of *Aspergillus nidulans* (hereafter *A. nidulans*), depending on various external and internal factors, can follow either the asexual or sexual developmental program to produce spores.

A fundamental step for the initiation of the asexual developmental program, which leads to the formation and maturation of the asexual spores (conidia) is the activation of the *brlA* (*bristle A*) promoter for an initial conidiation TF. This activation requires the derepression caused by SfgA (suppressor of *fluG*) and the displacement of the TFs NsdD (never in sexual development D) and VosA (viability of spores A) from the *brlA* promoter [1–3]. FluG (fluffy G) plays a major role for removing those repressive effects from the *brlA* promoter [4], hence, allowing the Flb (fluffy low brlA) regulators (FlbB, C, D and E) to directly activate the corresponding promoter [5–9]. FluG, SfgA and the Flbs are operating as upstream developmental activators (UDAs) for the *blrA* promoter [3]. Once the BrlA TF is expressed, it (directly and indirectly), controls a developmental cascade of genes, known as central developmental pathway (CDP) [3]. BrlA directly induces *abaA* (*abacus A*) expression encoding the regulator responsible for the middle phase of sporulation [8]. AbaA in turn activates, among other genes, *wetA* (*wet-white A*), that effects the expression of genes with roles at late conidiation stages [8,10–12]. The *stuA (stunted A),* encoding a helix-loop-helix TF, and *rgsA* (*regulator of G protein signaling*) genes are two additional components of the CDP. The expression of *stuA* is BrlA-dependent and a *stuA* deletion strain shows defects in the formation of conidiophores with only a few conidia. The RGS (regulator of G-protein signaling) proteins FlbA and RgsA are further promoting asexual development. In particular, FlbA alongside with FluG and the rest of the Flbs are required for the activation of *brlA* [13] and at the same time suppressing FadA, an inhibitor of asexual development [14]. RgsA is promoting conidia formation by attenuating the suppressive effects of GanB towards asexual growth [15].

The *A. nidulans* sexual developmental program is tightly controlled to produce the sexual reproductive spores (ascospores) in cleistothecia as enclosed fruiting bodies. The FadA (fluffy autolytic dominant A) protein, a member of the heterotrimeric G-protein (SfaD-GpgA-FadA), mediates signals from the plasma membrane to proteins (effectors) inside the cell [1]. When FadA is bound to GTP, it can initiate MAP-kinase signaling that leads to the activation of transcriptional regulators of the sexual development like NsdD and SteA (sterile12-like) [16]. NsdD (GATA-like TFs), NsdC (C2H2-type TF) and SteA (homeodomain-C2H2-zinc finger TF) are three major regulators of the sexual development [17–19]. Deletion of each of these genes results in a block in cleistothecia formation, highlighting their essential role during sexual development. Two additional transcriptional regulators important during the fruiting body formation are the Zn(II)2Cys6 TFs NosA (number of sexual spores) and RosA (repressor of sexual development) [20,21]. These two TFs have opposite roles, with NosA being an activator and RosA being a repressor of sexual development.

The velvet-domain proteins form a group of TFs with roles in the development and the secondary metabolism of numerous fungi. They were particularly studied in *A. nidulans* [22]. These proteins can interact with DNA through their velvet-domain, which has the same structural fold as the Rel domain of the mammalian NF-κB TF [23]. The four known velvet proteins of *A. nidulans* are VeA (velvet protein A), VelB (velvet-like B), VosA (viability of spores A) and VelC (velvet-like C). The ability of them to form homo- or hetero-dimers has been identified to be an important property. The velvet dimers define the shifting between developmental programs and regulate secondary metabolite synthesis [22,24–26].

In addition to the velvet regulators, the trimeric complex VapA-VipC-VapB plays an important role in the initiation of *A. nidulans* asexual development [27]. This complex is tethered to the plasma membrane by the zinc finger protein VapA. Upon perception of external signals, the methyltransferase heterodimer VipC-VapB is released from the membrane and can subsequently act in two ways. It interacts with VeA, hence, preventing it from entering the nucleus to induce the expression of genes promoting sexual development. The VipC-VapB dimer also enters the nucleus. Through their methytransferase activity the proteins decrease the H3K9me3 epigenetic mark in regulatory regions of genes encoding crucial asexual regulators such as BrlA (Bristle A) and AbaA (Abacus A). This initiates the asexual developmental program. The importance of the velvet proteins and the VapA-VipC-VapB trimeric complex in fungal development is known since years. However, the role of other potentially important developmental regulators and their possible interactions with the already established networks remains to be discovered.

Msn2 and Msn4 are functionally redundant Cys2His2 (C2H2) zinc-finger TFs implicated into stress response in *Saccharomyces cerevisiae* [28]. Their functions are related to the recognition of specific stress-response DNA elements (STREs), located in promoters of stress-related genes. The activation of these genes can be induced through binding of the Msn2 or Msn4 TF and results in a specific response to different stress stimuli at a time. Furthermore, Msn2 phosphorylation at minimum six serine residues by PKA (protein kinase A) appears to be crucial for the protein`s function in yeast [29]. A strain carrying an Msn2 allele with six serine-to-alanine substitutions (Msn2A6) shows defects in colony formation and a slow-growth phenotype when cultivated in a glucose-containing medium. Alongside with these findings, it has been also shown that lethality caused by the PKA deficiency can be rescued by Msn2 and Msn4 deletion. This highlights the antagonistic role of Msn2/4 and PKA in yeast and indicates that both TFs operate downstream of PKA [30]. The *A. nidulans* MsnA protein represents an orthologue of *S. cerevisiae* Msn2 or Msn4, which is also linked to stress responses. This indicates a conserved function of the protein in the fungal kingdom [31].

Another study focusing on *A. parasiticus* and *A. flavus* showed a function of MsnA in fungal development. Strains that carry the *msnA* deletion in both *Aspergillus* species produced more conidia than the wildtype. This suggests a repressive function of the protein on asexual development [32]. *A. parasiticus* MsnA is further linked to the regulation of secondary metabolite genes associated with the synthesis of the carcinogenic mycotoxin aflatoxin. This metabolite is part of fungal defense mechanisms. Therefore, MsnA from *A. parasiticus* is involved in a much broader cellular response than to oxidative stress [33]. Additionally, it has been demonstrated that BdMsn2 plays a critical role in the virulence of the fungus *Beauveria bassiana*. Specifically, it is important during penetration of the tick *R. microplus* and the synthesis of proteases to invade the cuticle [34]. *Metarhizium rileyi*, is another fungus with true filamentous growth, where the MrMsn2 TF is found to positively influence stress response, microsclerotia formation and virulence of the fungus [35]. The asexual conidiation of *Metarhizium acridum* can be shifted from normal conidiation after radial growth into microcycle conidiation which is referred to as yeast-like growth. MaMsn2, another homolog of yeast Msn2, was found to play a crucial role in retaining the pattern of normal conidiation [36]. Little is known regarding the contribution of MsnA to *A. nidulans* development, whereas the relationship of the protein and its paralogues to stress response has been extensively studied. Any potential interplay of MsnA and known key regulators of *A. nidulans* development remains elusive yet.

This study revealed that *A. nidulans* MsnA is a transcriptional regulator that shows a strong and direct *in vivo* binding to promoters of genes regulating asexual and sexual development. In addition, it fine tunes genes for appropriate secondary metabolism. MsnA directly controls the transcription of the epigenetic VapA-VipC-VapB methytransferase complex and therefore promotes fungal asexual development. Moreover, a novel genetic and molecular interplay between the fungal master regulators MsnA and VelB was discovered.

## Results

### The nuclear salt-stress regulator MsnA is required for *A. nidulans* development

Developmental processes are tightly connected to various stress signals in filamentous fungi. NADPH oxidases generate reactive oxygen species (ROS), which induce fungal sexual development [37,38]. Cellular complexes as the COP9 signalosome connect protein stability control [39] to transcriptional and metabolic responses as well as to hormones and oxidative stress protection and ultimately developmental programs [40].

**Fig 1:**
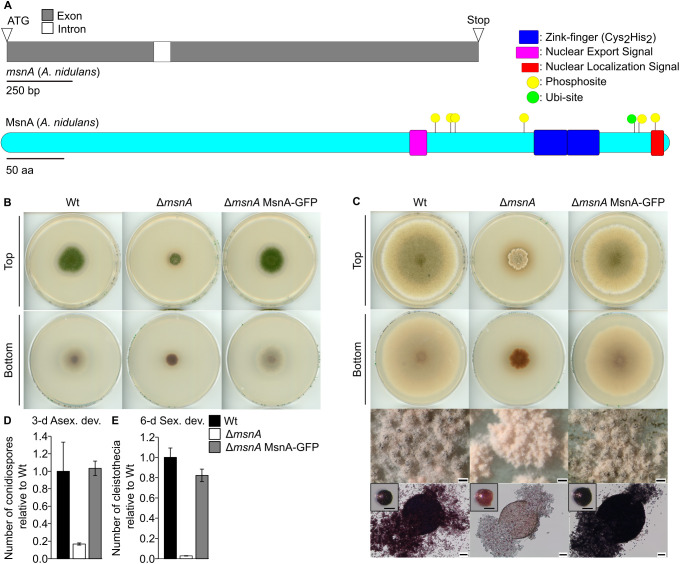
The MsnA stress-response TF is required for the development of *A. nidulans.* **(A)** Upper part open reading frame of *msnA* (AN1652), lower part protein domain analysis and predicted sites for post-translational modifications of MsnA. Blue rectangle = Zink-finger (Cys2His2) domain, purple rectangle = NES (Nuclear Export Signal), red rectangle = NLS (Nuclear Localized Signal), yellow circles = putative phosphosites and green circle = putative ubiquitination site. Phenotypical analysis of *A. nidulans* Δ*msnA* and complementation strain (Δ*msnA* MsnA-GFP), under (**B**) 3-d asexual (constant light) and (**C**) 6-d sexual (dark) promoting growth conditions. Scans derive from initial spots of 2000 conidia in the middle of the plate, which were then grow at 37 °C. Scale bars in overview photos (lower part of panel **C**) of many cleistothecia show a size of 200 µm, the bars shown in images of single intact cleistothecia represent 100 µm length, and the ones included in the images of broken cleistothecia represent 50 µm length. Quantification of (**D**) conidia and (**E**) cleistothecia of wildtype (Wt), Δ*msnA* mutant and complementation strain Δ*msnA* MsnA-GFP, after the indicative number of days, during asexual and sexual development. A total of 30,000 spores from each strain were spread equally (with glass beads) on each plate, and a minimum of three different plates were used per strain for the quantification.

The Msn2 and Msn4 Cys2His2 (C2H2) zinc finger TFs of the yeast *S. cerevisiae* or its counterpart MsnA in *A. nidulans* control various stress responses [28,31]. However, the molecular mechanisms linking stress response and developmental control or the contribution of MsnA (AN1652) to *A. nidulans* development are yet elusive. The *A. nidulans msnA* gene encodes an open reading frame of 1807 nucleotides interrupted by a single intron of 64 nucleotides (Fig 1A). The deducing 580 amino acids protein includes two Cys2His2 (C2H2)-type zinc finger DNA binding domains as found in the InterPro database [41], a nuclear localization signal (NLS), as predicted by the cNLS mapper tool [42], as well as a nuclear export signal (NES), as discovered by the LocNES algorithm [43] (Fig 1A). At least six phosphosites were predicted by the NetPhos tool [44] across the MsnA protein that might constitute direct kinase targets for phosphorylation. Additionally, there is at least one lysine residue predicted *in silico* by the UbiProber tool [45] representing a strong candidate site for a ubiquitination degradation signal recognized by the 26S proteasome.

An *msnA* deletion strain was generated to gain a better understanding of the MsnA contribution to *A. nidulans* growth and differentiation programs (Fig 1B and 1C). The deletion strain was complemented with a functional MsnA-GFP (green fluorescent protein) fusion encoding gene driven by its native promoter (complementation strain), which resulted in wildtype-like development. In contrast, the Δ*msnA* strain was severely affected in colony growth (Fig 1B and 1C). The deletion strain showed an altered colony morphology at the outer borders, which appear to be uneven. Colony growth was additionally much slower compared to wildtype and complementation strain, which is also visible upon longer incubation of the plates for 8 days under asexual or 12 days of sexual development inducing conditions (S1A and S1B Fig). The *msnA* deletion strain additionally showed changes in colony color, suggesting an altered secondary metabolism. The number of conidia produced after 3 days of asexual development is severely decreased in the deletion strain when compared to wildtype and complementation strain (Fig 1D). After 6 days of sexual development, the Δ*msnA* strain also produced reduced numbers of the closed sexual fruiting bodies. These cleistothecia, which are resting structures in the soil with protective and nursing Hülle cells that allowing survival during winter [22,46,47], appeared to be immature (Fig 1C and 1E). Incubation of the strains for 12 days, resulted in the formation of mature cleistothecia with sexual ascospores (S1B Fig), suggesting that resting structure production is delayed. These data support an important role for MsnA in *A. nidulans* growth and development.

Cellular localization of the MsnA-GFP was determined in submerged cultures of the complementation strain after 20 hours of vegetative growth. It was found that MsnA-GFP was clearly colocalized with the nuclear dye of Hoechst (S1C Fig). In summary, MsnA contributes to normal colony formation as well as sexual and asexual development.

### Genome-wide *in vivo* binding profiling of MsnA highlights its direct influence on various regulatory networks

**Fig 2:**
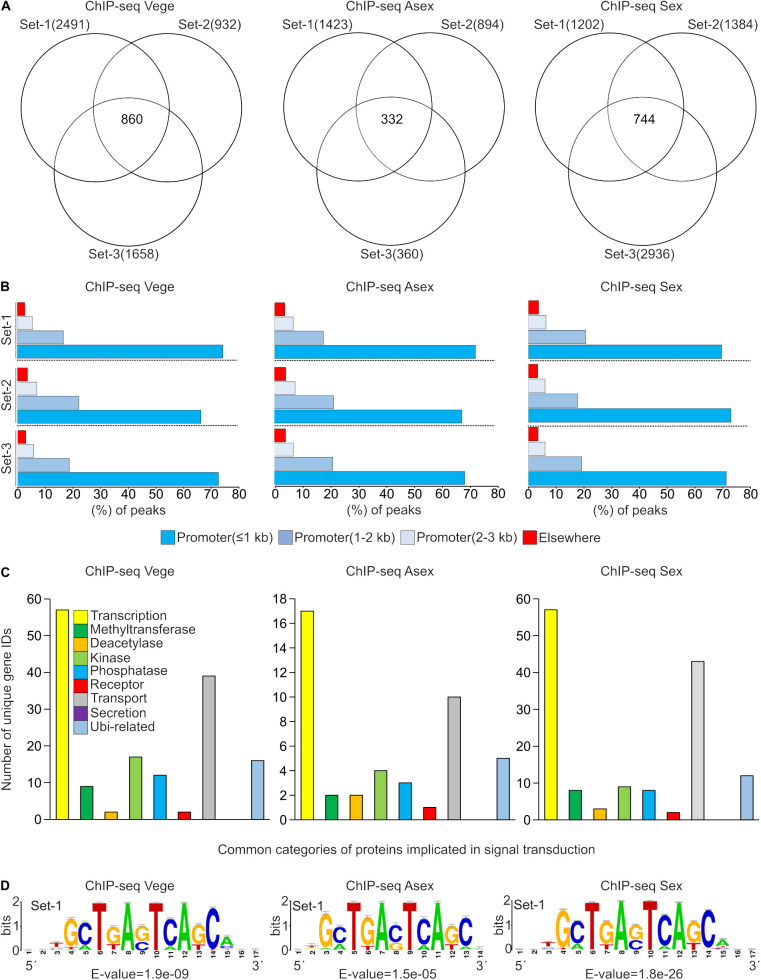
Genome-wide *in vivo* binding profile of MsnA-GFP in *A. nidulans.* **(A)** The Venn diagrams depict the overlap of three independent sets of the ChIP-seq analysis (MsnA-GFP versus input), performed in three different conditions, Vege growth, Asex and Sex development. For every independent set of analysis, the locus IDs derived after applying cut offs in fold enrichment (F.E.) ≥ 2.0, and with p-value < 0.05), associated with peaks (located in up to 3 kb promoter regions) found simultaneously in all three independent sets of the ChIP-seq samples, for each of the three ChIP-seqs. MsnA-GFP was detected by the GFP antibody and the corresponding input samples where used as negative control in all cases. **(B)** Bar charts show for both independent sets of the ChIP-seq analysis that around 70% of the statistically significant identified peaks are falling into promoter regions of genes spanning up to 1 kb from the TSS (Transcription Start Site). **(C)** Bar diagrams show the number genes encoding proteins of signal transduction processes. The numbers correspond to unique genes found after manual search in the sets of the common IDs identified from all three independent sets of analysis for each of the three ChIP-seq experiments. **(D)** Logos depict the top ranked *de novo* motifs, discovered by using the RSAT-peak-motif tool (https://rsat.eead.csic.es/plants/peak-motifs_form.cgi). For each independent set a group of the 100 bp sequences was used as input of the RSAT tool. Each of these sequences were located underneath the summit of the top 150 ChIP-seq peaks. All of those peaks were located into 3 kb promoter regions. A representative *de novo* discovered motif of one (out of three in total) independent set of analysis for each ChIP-seq is presented here, the rest are presented in S4 Fig.

The binding landscape of Msn2 from *S. cerevisiae*, particularly under stressful conditions has been studied *in vivo* in the past [48,49]*.* However, our current knowledge is restricted regarding the *in vivo* direct target genes of MsnA in *A. nidulans*. ChIP-seq experiments were performed by using the MsnA-GFP complementation strain with mycelia grown in three different conditions: 1) submerged culture grown for 20 hours at 37 °C vegetatively by shaking with a rotary shaker (hereafter Vege), 2) mycelia from submerged cultures grown for 20 hours at 37 °C by shaking with a rotary shaker, transferred to solid medium plates and incubated for another 6 hours under asexual growth condition, at 37 °C and light (hereafter Asex), 3) mycelia from submerged cultures grown for 20 hours at 37 °C by shaking with a rotary shaker, transferred to solid medium plates and incubated for another 8 hours under sexual growth condition, at 37 °C, dark and with restriction of O_2_ (hereafter Sex). Statistically significant peaks (p < 0.05) with fold enrichment (F.E.) ≥ 2.0, were identified as regions where there is significant alignment and enrichment of reads of the samples derived from the IPs with GFP-antibody of MsnA-GFP compared to the corresponding input control samples to which no antibody was applied. For each of the three ChIP-seq experiments, three independent sets of analysis were performed (each one consisting of a single distinct biological replicate with its input) to identify peaks that are fulfilling the previously mentioned cut offs and at the same time are located in regions up to 3 kb upstream from the transcription start sites (TSS) of genes. In total we identified for each ChIP-seq 860 locus IDs for Vege, 332 IDs for Asex and 744 IDs for Sex growth in the analysis of all three sets (Fig 2A and S4 Table). It was further examined, how the peaks identified from the ChIP-seqs, are distributed over different genetic elements of the genome. Around 70% of the identified peaks were found to be located to promoter regions of genes up to 3 kb from the TSS. The great majority of the binding events was mostly located in promoter regions spanning around 1 kb upstream from the nearby genes (Fig 2B).

The different types of proteins encoded by the genes, which are presumably targeted by MsnA, were determined and classified. A GO (Gene Ontology)-enrichment analysis was performed with the ShinyGO 0.82 webtool [50] with all genes found to be associated to MsnA within a range of 3 kb upstream from the corresponding transcription start site. The GO analysis showed that the most prominent and highly statistically significant target genes, found in the ChIP-seqs in all three different conditions, are coding for fungal proteins implicated in biological processes (BP) related to development, cell wall organization and various metabolic processes (S2 Fig). Search among nine protein categories, known to be associated with regulation or signal transduction, revealed top candidates related to transcription, phosphorylation or transport (Fig 2C). This corroborates a strong potential MsnA impact on the control of genes encoding proteins with direct implications in cellular signaling cascades.

The MsnA-associated DNA motifs found in all three ChIP-seq experiments with *A. nidulans* were compared for similarities to the previously described stress response element (STRE) sequences from *in vitro* [28] or *in vivo* [48] studies with *S. cerevisiae* (RGGGG motif). The RSAT-peak-motifs [51] webtool was employed. A *de novo* motif discovery was performed by using the 100 bp sequences located under the peak`s summits of the top 150 identified peaks (based to their fold enrichment).

The top-ranked *de novo* motif analysis identified a consensus DNA-binding motif of MsnA in *A. nidulans* (5´-GCTGAGTCAGC-3´) among all growth conditions tested (Vege, Asex and Sex) (Figs 2D and S4). Comparison of this MsnA motif found in our experiments with the STRE motif (RGGGG) from *S. cerevisiae*, showed that they do not share any similarities. The motif recognized by MsnA in *A. nidulans* is almost four times longer and with different base composition, compare to the STRE motif recognized by Msn2 from *S. cerevisiae* [48].

In summary, the ChIP-seq results from distinct developmental stages show that *A. nidulans* MsnA can recognize and subsequently be associated with a specific DNA motif, independently from the cultivation conditions. This motif is different from the *S. cerevisiae* STRE elements, where Msn2/4 was found to be associated with several stress responses. The *in vivo* binding of MsnA to its newly discovered elements potentially can influence the expression of genes that are associated with signal transduction pathways and fungal development.

### *A. nidulans* MsnA directly controls expression of genes encoding master regulators of fungal development

**Fig 3:**
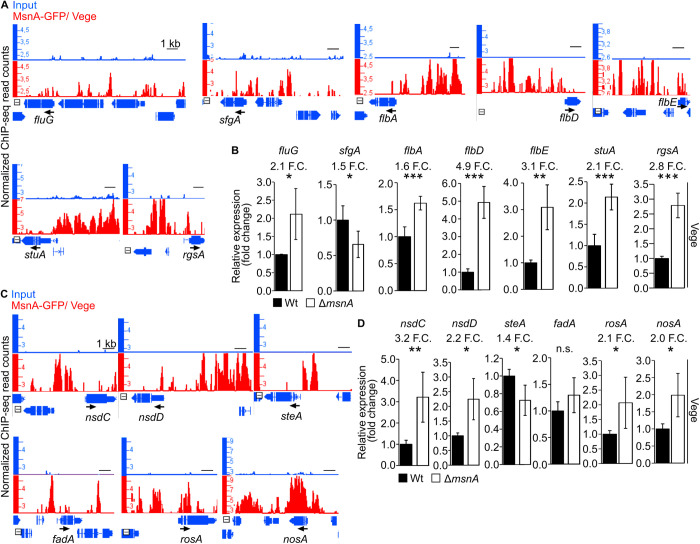
MsnA is associated *in vivo* with promoters and controls expression of genes encoding master regulators of *A. nidulans* asexual and sexual development, already during vegetative growth. Screen shots (**A** and **C**) from the Integrative Genome Browser (IGB) show peaks from Vege growth ChIP-seq data with MsnA-GFP located to the indicated promoters of regulatory genes. Small black horizontal arrows show the direction of the transcription of the corresponding gene below each screen shot. Further qRT-PCRs gene expression analyses (**B** and **D**) were performed, with RNA derived from wildtype (Wt) or Δ*msnA* strains grown under Vege (same conditions with the ChIP-seq experiment in **A** and **C**). At least three biological qRT-PCR replicates were performed per strain and per different time point, respectively. Each biological replicate includes a minimum of three technical replicates. Statistical differences for the gene expression data were performed by t-test, with *: p < 0.05, **: p < 0.01 and ***: p < 0.001, n.s.: not significant.

The MsnA-dependent developmental genetic network was investigated by searching for *in vivo* binding events of MsnA to promoters of genes regulating asexual or sexual development. *In vivo* binding of MsnA does not necessarily mean direct gene regulation. Therefore, comparative gene expression analysis by qRT-PCRs were performed between Δ*msnA* and wildtype to verify whether bindings correlate to changes in gene expression. Mycelia derived from Vege cultures were analyzed using the same conditions as for the ChIP-seq experiment. Binding of MsnA to the promoters of all regulatory genes tested, except *fadA,* correlated with significant changes in transcript expression under the same conditions. These findings corroborate that the MsnA regulates vegetative growth during the early stage when developmental competence is acquired (Fig 3). MsnA rather acts as repressor than as inducer of the identified genes during Vege growth, because 10 out of in total 12 differentially expressed genes showed increased expression in the Δ*msnA* strain compared to wildtype (Wt) (Fig 3B and 3D).

The impact of MsnA on genes encoding developmental regulators was further dissected by analysing early stages of asexual or sexual development. Gene expression was examined by qRT-PCRs with RNAs derived from wildtype (Wt) or Δ*msnA* strains, that were grown under Asex or Sex conditions respectively (S3B and S3D Fig). In most cases, the same genes as before were found to be regulated by MsnA and differentially expressed at different time points. Out of in total seven examined genes encoding asexual regulators, five were found to be direct *in vivo* targets of the MsnA while the fungus was growing under Asex conditions (S3A Fig). Moreover, with an exception of *flbA*, the rest of these genes followed the same expression pattern observed after Vege growth (Figs 3B and S3B). When focused on the sexual regulators, it was found that all six genes tested were found to be direct *in vivo* targets of MsnA under Sex growth conditions (S3C Fig). The expression of *rosA* was found to be unchanged under the same conditions (S3D Fig), although MsnA was able to be strongly associated with its promoter *in vivo* (S3C Fig). Additionally, MsnA was binding to the promoter region of *fadA* under Vege and Sexual growth. Its expression did not change in these conditions (Figs 3C, 3D, S3C and S3D). Noteworthy, the expression of *steA* followed opposite patterns during vegetative and sexual growth (Figs 3D and S3D). Nevertheless, at both conditions MsnA was able to clearly associate with the promoter of *stuA* (Figs 3C and S3C).

SclB (sclerotia-like B) is a zinc cluster (C6) TF with an important role in asexual development, secondary metabolism and oxidative stress response [52]. SclB is characterized as an activator of the asexual developmental program, controlling the expression of *brlA* and several other genes encoding regulatory proteins such as FluG, FlbC and FlbD, operating upstream from BrlA [52].

SclB activates genes, which were also found to be direct targets of MsnA, such as *flbD* and *fluG* (Figs 3A, 3B, S3A and S3B). Therefore, MsnA might also be able to control the expression of *sclB* and *brlA* as well. Multiple peaks in the MsnA ChIP-seq data at the promoter of *sclB* in close proximity with its TSS (*in vivo* binding positions of MsnA) were identified during vegetative and also during asexual growth (S3E Fig). When the promoter region of *brlA* was examined, we found that MsnA is able to bind mostly in a rather far away (tenths of kbs) upstream regions from the TSS of *blrA* during the vegetative growth. In contrast, Asex growth, MsnA was binding in close proximity of *brlA* in multiple occasions (S3E Fig). Another crucial regulator of asexual development is the WetA TF. Our data showed an *in vivo* binding of MsnA in close proximity of the *wetA* promoter, not only during vegetative but also during asexual growth conditions (S3E Fig).

Expression of *sclB*, *brlA* and *wetA* was investigated under the same conditions as for both ChIP-seqs with MsnA. Expression of all three genes was severely decreased in the Δ*msnA* strain during Vege growth (12.8 F.C. for the *sclB*, 3.8 F.C. for *brlA* and 3.7 F.C. for *wetA*) compared to the wildtype (Wt) strain (S3F Fig). In addition, the expression of *sclB* as well as of both functional overlapping *brlA* transcripts, *brlA*α and *brlA*β and *wetA* was examined at early stage of asexual development. Expression of *brlA* (*brlA*α and *brlA*β), *sclB* and *wetA* was strongly decreased under Asex development in the Δ*msnA* compared to wildtype strain (S3G Fig). These findings indicate a critical role of MsnA in positively regulating expression of *brlA*, *sclB* and *wetA* to support the asexual developmental program.

### MsnA directly controls fungal secondary metabolism during asexual development

Secondary metabolism as chemical language to communicate with the environment [22,53] is directly linked to development on a molecular level [25]. This includes the synthesis of chemical compounds that can be used as molecules with detrimental and often lethal effects against potential competitors or enemies of the fungus. Other bioactive molecules can either trigger or supress certain processes tightly associated with the developmental programs of the fungus [47,54].

The overall impact of MsnA regulator in the secondary metabolism of *A. nidulans* was examined. Secondary metabolites from wildtype (Wt) and Δ*msnA* strains grown under asexual conditions for 2 days were extracted and secondary metabolite profiles analyzed by LC-MS/MS were compared.

**Fig 4:**
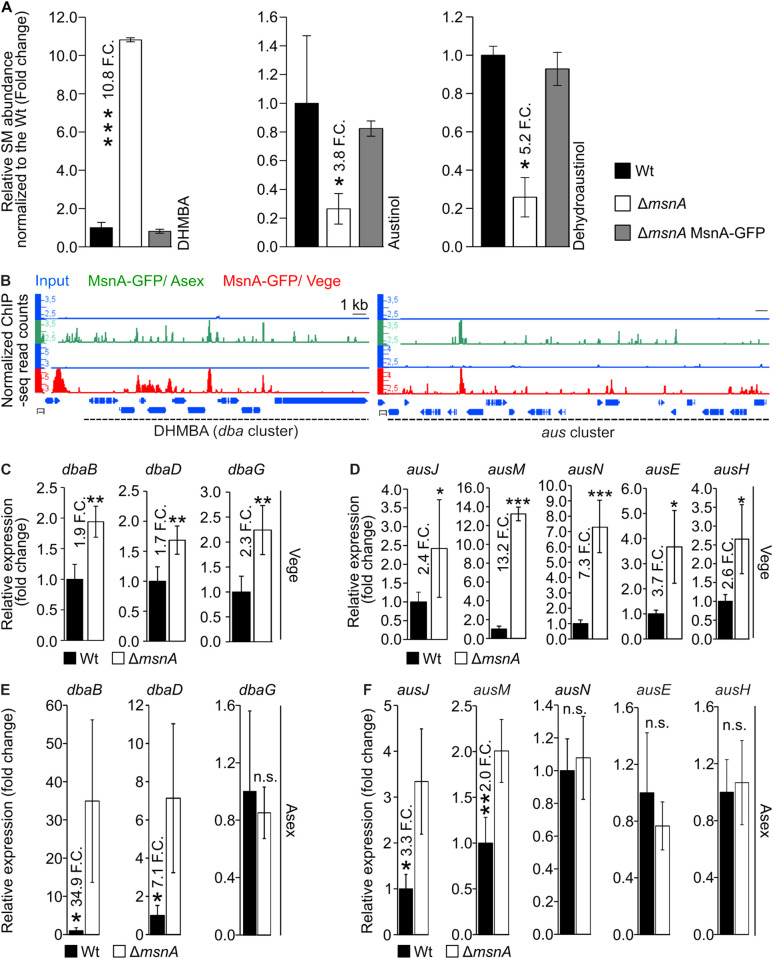
Fungal secondary metabolites associated with either defense or development of *A. nidulans* are controlled directly under asexual growth conditions by the stress regulator MsnA. Bar diagrams (**A**) depicting the relative abundance of secondary metabolites normalized towards wildtype; each strain is represented as the average and standard deviation of three replicates. Secondary metabolites were detected by LC-MS/MS with a charged aerosol detector (CAD) from extracts derived from the wildtype and the *msnA* deletion strain growing asexually for 2-d on minimal medium plates at 37 °C. **(B)** Screen shots from the Integrative Genome Browser (IGB) illustrating peaks from the ChIP-seq data with MsnA-GFP, located at promoters of genes of the *dba* and *aus* gene clusters, derived from mycelia either growing under Vege (red tracks) or Asex conditions (green tracks) upon solid medium plates; the blue tracks indicate the corresponding inputs (negative controls) at each case. Gene expression was analyzed by qRT-PCR, for genes of the *dba* (**C** and **E**) and *aus* (**D** and **F**) gene clusters. RNAs (for **C** and **D**) derived from mycelia of wildtype and Δ*msnA* strain, grown under Vege conditions. RNAs (for **E** and **F**) came from mycelia of the same two strains, growing Asex conditions. The gene expression data presented in this figure represent average values and standard deviations of at least three biological replicates with each deriving from at least three technical replicates; Statistics made by Student’s t test: *P ≤ 0.05, **P ≤ 0.01 and ***P ≤ 0.01, n.s.: not significant.

Austinol and dehydroaustinol were severely reduced in the absence of MsnA compared to wildtype. Especially dehydroaustinol together with diorcinol, was found to restore defects of the Δ*fluG* strain in sporulation. Hence, it was concluded that it promotes asexual development [55] (Fig 4A). Moreover, the antimicrobial DHMBA (2,4-dihydroxy-3-methyl-6-(2-oxopropyl) benzaldehyde [56] was increased approximately eleven-fold in the *msnA* deletion strain compared to wildtype during the same growth conditions (Fig 4A). DHMBA has shown to possess antimicrobial activity against the Gram-positive bacterium *Micrococcus luteus* in agar diffusion tests. In these tests, the inhibition zone that was generated by a fungal strain that was producing increased amounts of the corresponding SM was measured. In conclusion, these data support multiple control functions of MsnA to adjust defense and signal secondary metabolite levels for supporting asexual fungal development. Secondary metabolite profiles of Δ*msnA* versus the wildtype strain were additionally examined during vegetative and sexual growth of the fungus, but no significant differences were observed for any known metabolite.

The secondary metabolite profiles of the Δ*msnA* mutant strain revealed an important direct or indirect MsnA control function of the enzyme synthesis of several known secondary metabolites. The ChIP-seq data were searched for peaks that are potentially located to the promoters of the corresponding secondary metabolite gene clusters of differentially synthesized molecules. Peaks were identified among the genes of the cluster that are associated with the formation of DHMBA, austinol and dehydroaustinol, at Vege and Asex growth (Fig 4B).

Subsequently, it was investigated if peaks from the ChIP-seq data, found to be associated with secondary metabolite genes, are able to directly control the expression of genes for the synthesis of DHMBA, austinol and dehydroaustinol. Gene expression of wildtype and the Δ*msnA* strains was compared during vegetative growth. The ChIP-seq peaks found inside the *dba* gene cluster (Fig 4B) support the hypothesis that MsnA exerts a direct transcriptional control of several DHMBA cluster genes. A gene expression study confirmed that out of the nine genes that constitute the *dba* cluster in total, at least three showed significant changes in gene expression in the Δ*msnA* strain compared to wildtype (Fig 4C). The changes in gene expression indicate a clear repressive role of MsnA, which is in line with the increase of the DHMBA synthesis in the Δ*msnA* strain (Fig 4A).

Our study has also shown that there is a strong involvement of MsnA to the synthesis of specific metabolites such as austinol/dehydroaustinol during asexual growth of the fungus (Fig 4A). These results were corroborated further by ChIP-seq data, from asexual and vegetative growth as well. These data showed that there is at least one strong peak located to a putative promoter region of genes of the *aus* cluster and several other smaller peaks spread along the whole *aus* cluster as well (Fig 4B). Therefore, we examined the expression of genes that belong to the *aus* cluster when the fungus was grown vegetatively. The expression of the specific genes (*ausJ*, *M*, *N*, *E* and *H*) from the *aus* cluster was found to be strongly increased in the Δ*msnA* strain (Fig 4D). Deletion of these genes leads to abolishment of austinol and dehydroaustinol synthesis [57]. These results underline the prominent direct regulatory role of MsnA towards the expression of genes essential for the synthesis of austinol and dehydroaustinol.

When the expression profile of the previously examined genes was examined under Asex development, the results were found to be slightly different. From the three genes of the *dba* cluster that were found to be differentially expressed during vegetative growth (*dbaB*, *dbaD* and *dbaG*), two maintained their expression profile after shifting to Asex growth, but *dbaG* was not differentially expressed anymore (Fig 4E). Moreover, under the asexual growth conditions from in total five *aus* genes, that found to be differentially expressed during vegetative growth only two (*ausJ* and *ausM*) kept the same expression pattern and the rest (*ausN*, *ausE* and *ausH*) was not found to be differentially expressed (Fig 4F). In conclusion, MsnA directly controls secondary metabolite clusters expression and subsequent production of secondary metabolites associated with fungal development and defense.

### An uncharacterized transcriptional circuit among MsnA and the velvet master regulators

The impact of MsnA on *A. nidulans* development has to be embedded into the corresponding regulatory networks including other known key regulators of differentiation as well as the response elements to external signals. Velvet domain proteins share a similar fold for DNA binding and dimerization which is reminiscent to mammalian NF-kB and are key regulators of fungal development [23]. The four velvet- domain proteins VeA, VelB, VelC and VosA can form various homo- and heterodimers with distinct function in fungal development. VelB can form a heterodimer with VeA [25], but can also associate with VosA [24].

**Fig 5:**
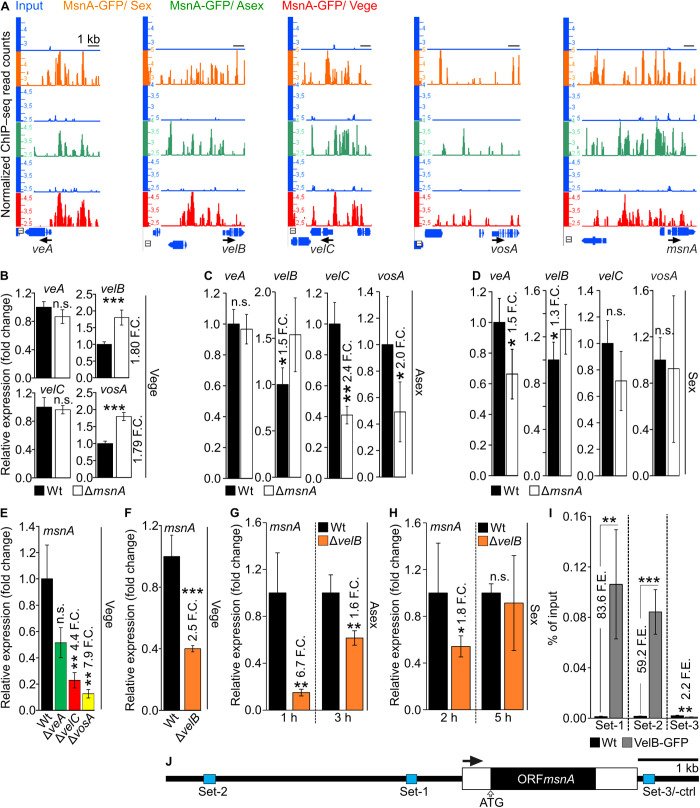
Stress regulator MsnA and velvet domain developmental regulators can reciprocally transcriptionally control each other. Screen shots from the Integrative Genome Browser (IGB) (**A**) showing peaks (binding events of MsnA-GFP) from the ChIP-seq data under Vege (red tracks), Asex (green tracks) and Sex (orange tracks) growth, where MsnA-GFP associated with promoters of the velvet genes. **(B)** Gene expression analyses of the *veA*, *velB*, *velC* and *vosA* via qRT-PCRs, with mycelia derived from submerged cultures of wildtype (Wt) and the deletion strains Δ*msnA*, grown under Vege conditions. Gene expression analysis for the velvets, with RNAs derived from Wt and Δ*msnA* strain grown **(C)** Asex or **(D)** Sex growth conditions. Gene expression analyses of *msnA* from mycelia of wildtype (Wt), Δ*veA*, Δ*velC* Δ*vosA* and Δ*velB*, grown Vege conditions **(E** and **F)**. Gene expression profiling of *msnA,* with RNAs derived from wildtype (Wt) and Δ*velB* strains growing under **(G)** Asex and **(H)** Sex growth conditions. ChIP-qRT-PCRs (**I**) for examining the *in vivo* binding of the VelB-GFP (expressed under its native promoter) to the promoter of *msnA*. The cartoon of the panel (**J**) depicts with blue boxes the different regions of DNA where the binding of VelB-GFP was tested by using distinct sets of primers at a case; set-1 and set-2 constitute regions where VelB-GFP is highly associated with, and set-3 is a region downstream of the *msnA* that was used as a negative control. The black arrow, just above the gene structure indicates the direction of the transcription. For all the gene expression results and the ChIP-qRT-PCRs, presented data are averages and standard deviations of at least three biological replicates with each deriving from at least three technical replicates; Statistics made by Student’s t test: *P ≤ 0.05, **P ≤ 0.01 and ***P ≤ 0.01, n.s.: not significant.

The initial approach was to examine whether MsnA controls expression of any of the velvet genes directly. The MsnA-GFP ChIP-seq data, from all tested conditions, Vege, Asex and Sex growth, were examined for peaks on the promoters of the velvet genes. Strong multiple peaks were discovered in the promoters of *veA*, *velB* and *velC* (Fig 5A). The promoter of *vosA* was found to be far less occupied by bindings of the MsnA, compared with the other velvet promoters. However, an association of MsnA to few sites of the *vosA* promoter was detected as well (Fig 5A).

A gene expression analysis was performed with mycelia grown under Vege growth. It was examined whether presence or absence of MsnA affects gene velvet domain protein expression. MsnA primarily represses transcription of the *velB* as well as the *vosA* gene (Fig 5B). The *in vivo* binding of MsnA to the *veA* promoter, does not seem to induce differential expression of this gene at this particular time point and growth condition (Fig 5A and 5B). Moreover, the rather small peaks detected under vegetative growth at the promoter of *vosA*, probably contribute to the differential expression of *vosA* in the Δ*msnA* strain compared to wildtype (Fig 5B). In sum, the *in vivo* association of MsnA with the promoters of *veA*, *velB*, *velC* and to a lesser degree *vosA* during vegetative growth leads to the differential expression of *velB* and *vosA* only.

A comparative gene expression analysis of RNA derived from Vege mycelia, was conducted to examine whether velvets can affect the expression of *msnA*. This analysis revealed that the deletion strains of *velC* and *vosA*, but not *veA* showed a severely reduced expression of the *msnA* gene with the most prominent impact in the *vosA* deletion strain (Fig 5E). This suggests that VelC or VosA are required for the production of sufficient *msnA* transcripts either by a direct or indirect molecular mechanism.

A potential regulatory circuit between VelB and MsnA was examined in more detail, because the expression of the *velB* transcript is directly controlled by MsnA. Gene expression analysis with the wildtype and the *velB* deletion strain was not only performed with mycelia grown under vegetative conditions but also with mycelia transferred onto solid medium after vegetative growth and then further incubated under conditions favoring asexual or sexual development. Expression of *msnA* was found to be significantly repressed in the *velB* deletion strain (Δ*velB*) (Fig 5F–5H). These results revealed that VelB was able to induce the expression of *msnA* during vegetative, asexual and sexual growth, which supports a mutual control between both TFs.

VelB continues promoting the expression of *msnA* transcripts after the shift from vegetative growth to illuminated growth on plate promoting asexual development for up to three hours with a peak after one hour (Fig 5G). The influence of VelB on the expression of *msnA* during sexual development under dark conditions on solid media is evident at the onset of this developmental program but completely diminishes within five hours (Fig 5H).

Lastly, since we discovered the VelB regulatory function on *msnA* expression, it was further examined whether this is an indirect control or based on a direct VelB *in vivo* binding to the *msnA* promoter region. A ChIP experiment was performed with mycelia from the VelB-GFP and the wildtype strain grown under Vege conditions. This revealed two *msnA* promoter positions (Fig 5I and 5J, set-1 and set-2) proximal to the TSS with strong *in vivo* VelB association in comparison to the control ChIP-qRT-PCR to a downstream region of *msnA*, where the ChIPed DNA of the IP with VelB-GFP was lower than the corresponding IP with wildtype (Fig 5I and 5J, set-3).

In summary, a novel mutually controlled genetic network between two key regulators of stress response (MsnA) and fungal development (VelB) was discovered. MsnA is also strongly associated *in vivo* with its own promoter (Fig 5A), which supports an additional level of autoregulation to further influence its own transcription. VelB and MsnA do not only control their own expression but also the expression of other key control genes for distinct developmental fungal programs through this network.

### *msnA* and *velB* show different genetic influence during fungal development

**Fig 6:**
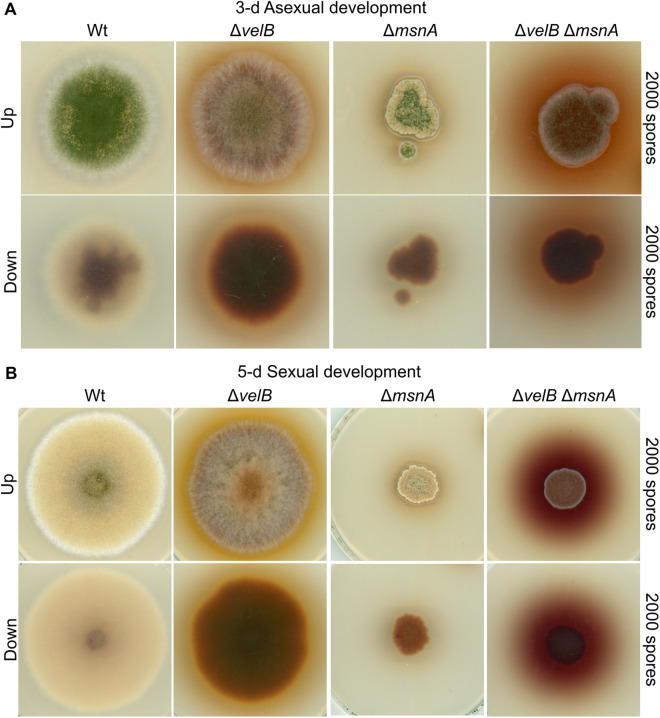
The double mutant of *msnA* with the *velB* shows an additive phenotype. Scans of different strains grown under 3-d asexual (**A**) and 5-d sexual (**B**) conditions represent the phenotypes of the single and the corresponding double mutants of *msnA* and *velB*. For both phenotypical assays, spores from the depicted strains were initially spotted (2000 spores/plate) on minimal medium plates. Subsequently scans made from top of the plates after the indicated period of time, under either asexual or sexual growth conditions. Scans from the bottom of the plates highlight differences in the overall colorations of the fungal colonies, which is an indicator for changes secondary metabolism of the corresponding strains.

Genetic interactions between the *msnA* and *velB* genes were further explored due to the molecular regulatory interplay discovered among MsnA and VelB. A double deletion strain of *msnA* and *velB* was generated. Under asexual growth conditions the single mutants Δ*msnA* and Δ*velB* showed distinct phenotypes, regarding conidiospores formation and secondary metabolism (as can be observed macroscopically from the bottom of the plates) (Fig 6A). The phenotype of the Δ*velB* Δ*msnA* double deletion strain is different, because it is a rather additive phenotype of both genes. More specifically, the examination of the *velB msnA* double deletion strain under conditions that favor sexual development of the fungus showed prominent and distinctive phenotypes compared to the corresponding single mutants (Fig 6B). For example, *msnA* controls radial colony growth and *velB* strain pigmentation. In summary, MsnA and VelB do not only mutually control their transcriptional regulation (Fig 5) but further control distinct phenotypical features under either asexual or sexual growth conditions.

### MsnA controlled salt-stress response is independent of VelB

**Fig 7:**
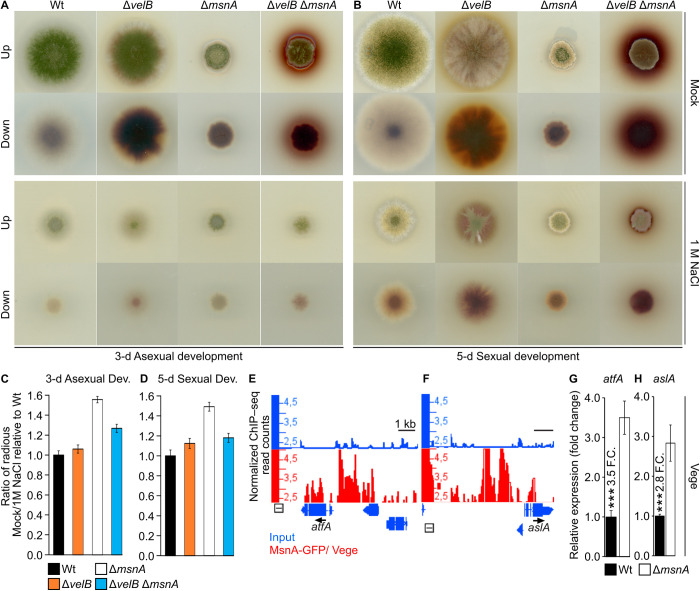
MsnA, but not VelB, mediates salt-stress response in *A. nidulans.* Phenotypical characterization of the Δ*msnA*, Δ*velB* and their corresponding double mutants under salt-stress. Scans of different strains under mock and 1 M NaCl treatment accordingly for 3-d asexual (**A**) and 5-d sexual (**B**) development. Initially 2000 spores were spotted in the middle of each medium plate. Bar diagrams show the ratio of the diameters of the colonies for different strains without (Mock) and with (1 M NaCl) the application of the salt treatment during 3-d asexual (**C**) and 5-d sexual (**D**) development; in both conditions data were normalized to the Wt’s Mock/Salt ratio. Presented data are averages and standard deviations of four biological replicates per each strain (under mock and treated conditions). The screen shots from the Integrative Genome Browser (IGB) (**E** and **F**) show peaks from the MsnA-GFP Vege growth conditions ChIP-seq data, where MsnA is associated *in vivo* with promoters of *atfA* and *aslA*, encoding for known regulators of the salt stress in *A. nidulans*. Gene expression analyses of *atfA* and *aslA* (**G** and **H**)*,* under Vege growth (without salt-stress treatment), via qRT-PCRs, showing the strong repressive role of MsnA to the *atfA* and *aslA* gene expression. Presented gene expression data are averages and standard deviations of at least three biological replicates with each deriving by at least three technical replicates; Statistics made by Student’s t test: ***P ≤ 0.01.

MsnA is a central regulator in the salt-stress response of *A. nidulans* [31]. Based on our previous findings regarding the interaction of MsnA with VelB on a cross-regulatory level, it was hypothesized that such interactions might also play a role when the fungus is growing under salt-stress conditions. Therefore, the potential mutual control of VelB and MsnA was analyzed under salt stress. Initially, single or double deletion strains of *msnA* and *velB* were cultivated on solid medium plates containing 1 M NaCl under conditions that either induce asexual or sexual development. Diameters of stressed and non-stressed colonies of all the strains were measured and the ratios between mock and treatment were calculated. The salt treatment resulted in a retardation in colony growth for all strains (Fig 7A and 7B). The quantification of this effect showed that only strains carrying an *msnA* deletion showed striking differences compared to wildtype (Wt) during asexual and sexual development (Fig 7C and 7D). In fact, presence of the *velB* deletion did not contribute to the fungal salt-stress response, even when combined with Δ*msnA*. Under the given stress conditions the double mutant of *msnA* and *velB* showed an additive phenotype of the single mutants during asexual and the sexual growth (Fig 7A–7D). This suggests that under the given growth and stress conditions *msnA* and *velB* are regulating clearly different phenotypical features. In sum, the application of salt stress to the corresponding *velB* and *msnA* deletion strains revealed a positive implication of MsnA but not of VelB on fungal growth, independently of the fungal developmental program. Little is known about the regulation of salt-stress genes by the MsnA in *A. nidulans*, whereas the binding profiling of Msn2 from *S. cerevisiae* (as ortholog of MsnA) during oxidative stress has been studied years ago [48,49]. It was initially hypothesized that *A. nidulans* MsnA might be able to occupy promoters of major regulators of the salt-stress signaling, even without any particular salt conditions. The ChIP-seq data generated under vegetative growth conditions for the current study were searched for *in vivo* binding events of MsnA to promoters of known salt-stress regulators. The bZIP-type AtfA (Activating transcription factor A) and the C2H2-type zinc finger AslA (Asexual differentiation with low-level conidiation) TFs are implicated in salt-stress response of *A. nidulans* [58,59]. The promoters of the *atfA* and *aslA* genes, were found to be strongly occupied by MsnA (Fig 7E and 7F). A subsequent gene expression analysis of *atfA* and *aslA* in the Δ*msnA* and Wt strains from mycelia grown under vegetative conditions was performed. The results from this analysis showed that *atfA* and *aslA* expression was strongly increased in the Δ*msnA* compared to wildtype strain (Fig 7G and 7H). These results further confirmed that those *in vivo* regulatory interactions of MsnA with the promoter of *atfA* and *aslA* are of biological significance. All these findings together propose that in the absence of salt-stress, MsnA even prevents the unreasonable initiation of a salt-stress response by attenuating the expression of the *atfA* and *aslA* genes coding for salt-response regulators.

### MsnA directly regulates genes encoding methyltransferases that play major roles in development

**Fig 8:**
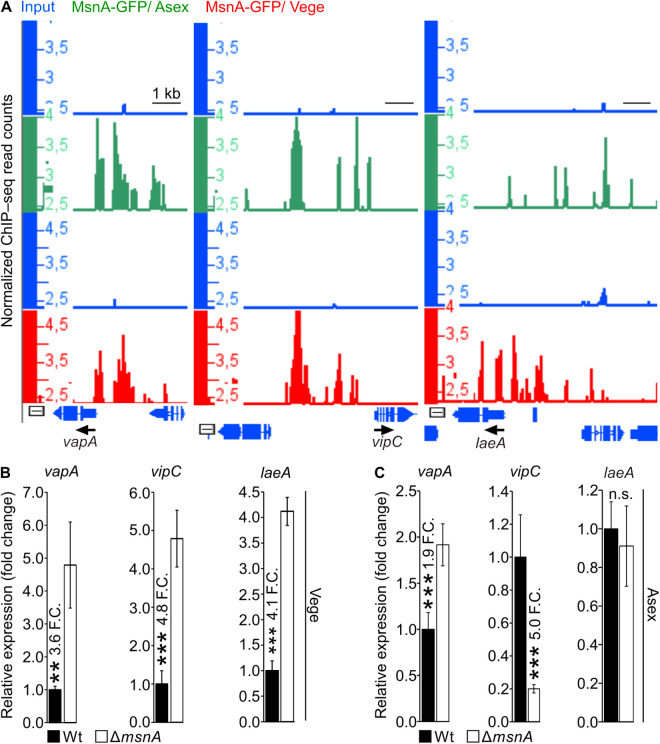
MsnA is associated *in vivo* to the promoters of *laeA* methyltranferase and the *vapA* and *vipC* genes encoding for parts of the VapA-VipC-VapB (asexual development promoting) methyltransferase complex. Screen shots (**A**) from the Integrative Genome Browser (IGB) depicting the presence of MsnA-GFP to the *vapA, vipC* and *laeA* promoter regions. Gene expression profiles (**B** and **C**) by qRT-PCR, for all the genes depicted in (**A**). (**B**) RNAs derived from mycelia of wildtype (Wt) or Δ*msnA* strain, growing under Vege conditions. Expression profiling (**C**) of *vapA*, *vipC* and *laeA* derived from mycelia of wildtype (Wt) and Δ*msnA* strains growing Asex growth for the indicative time points. All gene expression data presented are average values and standard deviations of at least three biological replicates with each deriving by at least three technical replicates; Statistics made by Student’s t test: *P ≤ 0.05, **P ≤ 0.01 and ***P ≤ 0.01, n.s.: not significant.

Sexual development is promoted by the trimeric complex of two velvet proteins, VeA and VelB, with the methyltransferase LaeA [25]. Asexual development is favored by the restriction of the VeA from the nucleus. The VapA-VipC-VapB complex is tethered to the plasma membrane via VapA in the dark, which favors sexual development. In light, the VipC-VapB methyltransferase complex is released, and it restricts the entry of VeA into the nucleus through physical interactions. VipC-VapB further enters the nucleus where it epigenetically affects the methylation status of histones, which leads to changes in the expression of genes that promote asexual development [27]. MsnA plays a major role in asexual development, alongside with its molecular interplay with the velvet domain proteins. It was examined whether MsnA has a direct role in the regulation of these methyltransferases, which control either the asexual or the sexual developmental program of *A. nidulans*. Therefore, the MsnA ChIP-seq data, derived from Vege and Asex grown mycelia, were analyzed for peaks nearby the genes of the methyltransferases. Peaks were detected proximal to the gene of the Zinc finger VapA membrane protein, the VipC and the LaeA methytransferase as well (Fig 8A). Next, we wanted to verify if these *in vivo* binding events of MsnA to the promoters of *vapA*, *vipC* and *laeA* can influence the expression of these genes. A gene expression analysis was performed with wildtype and Δ*msnA* strains. Gene expression profiles from Vege grown strains and mycelium additionally grown on solid medium plates under Asex development were analyzed. The expression of *vapA*, *vipC* and *laeA* was increased in the Δ*msnA* strain compared to the wildtype (Fig 8B), validating the binding of MsnA to the promoters of these genes (Fig 8A). When the expression of *vapA*, *vipC* and *laeA* was followed during early stages of the asexual development, a different pattern emerged, particularly for *vipC* and *laeA* expression. The expression of *vapA* was increased in the Δ*msnA* strain (Fig 8C) similar as was observed during vegetative growth (Fig 8B). In contrast, the expression of *vipC* was found to be repressed by MsnA during vegetative growth (Fig 8B), whereas it was induced by MsnA under asexual growth conditions (Fig 8C). Moreover, *laeA* was found not to be differentially expressed during early asexual development (Fig 8C). In summary, MsnA has a new and previously elusive role as a transcriptional regulator of complexes from the velvet and methyltransferase families with essential and established roles during the two distinct fungal developmental programs.

## Discussion

During its life span, *A. nidulans* has to respond to a variety of internal and external stimuli to coordinate and promote its development. This includes the production of spores, which secure fungal distribution and survival. Several characterized, but also plenty of yet uncharacterized TFs play major roles in this essential orchestration. Currently there are examples where the role of a specific TF in various processes has been elucidated but its direct implication (for example, which are the direct genome-wide target genes) during development remains elusive. *A. nidulans* MsnA falls into this category of TFs. As a C2H2-type zinc-finger TF, MsnA, has been linked to stress responses such as salt/osmotic, temperature and hydrogen peroxide/oxidative (H_2_O_2_) stress [31,32]. There is lack of knowledge regarding the roles of MsnA during fungal development, specifically on a genome-wide scale. Here we show that MsnA plays a major role in the development of *A. nidulans* under non-stress conditions. This is happening mainly in two ways: i) MsnA directly binds *in vivo* to promoters of genes encoding master regulators under different growth conditions including vegetative, asexual and sexual growth ii) MsnA fine tunes secondary metabolism, specifically during the asexual development of the fungus.

The evidence provided by the current literature, supporting a direct implication of MsnA in *A. nidulans* development under non-stress conditions are quite restricted. The *msnA* deletion strain displays a severe growth restriction of the colony when grown in light. Nevertheless, the ability of the strain to form developmental structures was not further investigated or quantified [60]. Our data show that the deletion of *msnA* causes a very strong reduction in colony size, the numbers of asexual spores (conidia) and the number of the sexual structures (cleistothecia) that carry the sexual spores (ascospores) (Fig 1B–1E). Nevertheless, apart from the developmental delay of the Δ*msnA* strain compared to wildtype, both developmental programs remain functional and are completed in the deletion strain (S1A and S1B Fig).

In yeast the preference of Msn2 to associate with certain regions of the genome under stress has already been studied the last decade. It has been shown that there is a specific dynamic interplay among Msn2 binding and the nucleosome occupancy that is crucial for the activation or (and) the repression of specific genes nearby [48,49]. However, in *A. nidulans*, up to now, neither a genome-wide *in vivo* occupancy preference of MsnA was studied, nor the overall concept that MsnA, can operate as a TF. Even though it has been postulated that MsnA can control the expression of genes, there is hardly any data in literature where *A. nidulans* MsnA is directly associated with a promoter of a gene *in vivo*. MsnA can interact with the *mstB* promoter *in vitro*. The MstB protein is presumably involved in transportation of monosaccharides that are required during sexual development [61]. In the related fungus *A. parasiticus*, MsnA is associated with specific promoters of genes involved in the synthesis of aflatoxin and stress response, which has been shown by *in vitro* binding assays [33]. Here we show that MsnA is a nuclear localized protein, able to interact with many promoters of genes, across the genome of *A. nidulans*, under distinct growth conditions *in vivo*. Some of these genes encode major regulators of asexual or sexual development (Figs 3 and S3). In almost all cases, MsnA was directly binding near the TSS of these genes. *A. nidulans* MsnA does not share a similar DNA recognition motif (stress response element, STRE) with yeast Msn1 [48]. Instead, it is able to recognize a larger and differently composed DNA sequence in the gene promoter regions it is associated with *in vivo*. This finding was consisted in all different growth conditions tested (Vege, Asex and Sex). MsnA is located to the promoters of genes via its direct molecular interaction with these elements. As a result, gene expression is influenced immediately (Figs 3 and S3). In sum, our findings highlight the role of MsnA as an important regulator for *A. nidulans* development, which can influence the expression of several known development-related genes directly, hence promoting both developmental programs.

Data, where orthologs of MsnA from other filamentous fungi (*A. parasiticus* and *A. flavus*) showed with a regulatory effect on secondary metabolism was related to the production aflatoxins and kojic acid [32]. Our broader approach elucidated that MsnA is directly implicated in the synthesis of secondary metabolites, specifically under the asexual growth that are not only related to defense mechanisms of the fungus (such as DHMBA) but also to the regulation of development (such as austinol and dehydroaustinol) (Fig 4). Therefore, these data underline a novel aspect of MsnA, acting as a major hub where stress signaling, secondary metabolism and development converge and are coordinated, to help the fungus coping with different environmental stimuli. However, discrepancies observed in phenotypes of Δ*msnA*, compared to Wt, and the expression of genes related to the *aus* cluster, might be caused by other types of molecular interactions, mediated by MsnA, which were possibly not elucidated by our experiments.

The coordination of developmental processes is rarely an act of a single regulator. Instead, it often involves collaborative work of several key regulators, which can collectively orchestrate the necessary actions required for progression of fungal development based on external and internal stimuli. According to this concept, it was discovered that MsnA is collaborating with other key regulators, to progress fungal growth. A regulatory circuit between MsnA and velvet regulators was disclosed. All velvet proteins, except VeA, were able to affect *msnA* expression. MsnA was able to associate under all tested growth conditions with the promoters of the velvet genes *in vivo*. Although, MsnA was far more prominently attached to the promoter regions of *veA*, *velB* and *velC,* it was also found to weakly interact with the promoter of *vosA* (Fig 5). However, these *in vivo* binding events of MsnA had various impacts on the velvet genes expression, that were depending on the growth conditions tested. It was further shown that MsnA and VelB can mutually control each other`s expression through *in vivo* association with their promoters (Fig 5). This molecular regulatory circuit among MsnA and VelB drove us to study the genetic interplay between the two genes. We discovered that *msnA* and *velB* have an additive genetic interaction, with each protein being responsible for distinct phenotypical characteristics (Fig 6). In sum, the novel molecular regulatory circuit among MsnA and VelB is further enhanced and supported by additional genetic data that showed the two regulators, to contribute mostly in different aspects the fungal development. Additionally, it was shown that an autoregulatory transcription loop might make MsnA to control its own expression (Fig 5A).

The VapA-VipC-VapB methytrasferase complex, is known for transmitting the signal that induces asexual development in light from the plasma membrane into the nucleus [27]. This process involves release of the VipC-VapB heterodimer from the plasma membrane, where is tethered via VapA. The dimer subsequently interacts with VeA, which keeps it out of the nucleus, hence, makes it unable to interact with the nuclear LaeA methyltransferase to initiate sexual development in light [27]. The reciprocal regulatory interplay between MsnA and the velvet proteins, alongside with the relationship of VipC with VeA [27], led to the hypothesis that there might be a link between MsnA and VapA-VipC-VapB complex. Our *in vivo* binding experiments showed that MsnA was strongly associated with the promoters of *vapA*, *vipC* and *laeA* (Fig 8A). These specific DNA-protein interactions were found to influence the expression of *vapA*, *vipC* and *laeA* directly. In fact, all three genes were repressed by MsnA during vegetative growth. However, the expression profiles of *vipC* and *laeA* changed during asexual growth; *vipC* was found to be induced and *laeA* was not differentially expressed by MsnA (Fig 8C). Based on the current model the interplay between VipA-VipC-VapB and VeA-VelB-LaeA [25,27] complexes are balancing the developmental programs of the fungus. Our findings support this model by revealing another layer of transcriptional regulation governed by MsnA, towards components of both complexes.

The role of MsnA in stress signaling across many different fungal species is relatively well-established. Various stress stimuli can trigger *msnA* expression, or its paralogs from other fungi, among them salt/osmotic, oxidative and heat stress [31,32,62]. The discovery of the molecular and genetic network between MsnA and VelB, alongside with the lack of knowledge on the potential implication of VelB in salt signaling, formed a new hypothesis. In *A. nidulans* VelB might be able to mediate a salt-stress response together with MsnA. However, our data indicate that VelB does not contribute to a salt-stress response. Moreover, strains that lack *msnA* show a strong retardation in colony formation compared to wildtype during growth with 1 M NaCl. Investigation of the double *velB* and *msnA* double deletion strain, indicated that each TFs confers a different contribution to colony growth and fungal secondary metabolism under salt-stress conditions. These findings would potentially point to an additive rather an than epistatic relationship among the two regulators (Fig 7A–7D). However, data from *A. niger* showed that there was no significant effect on colony formation of the *msnA* deletion grown with 1 M NaCl compared to wildtype [63]. This might suggest that the important role of *A. nidulans* MsnA during salt stress is not conserved in *A. niger*.

Our study revealed another novel role of MsnA related to the control of stress response. It was found that genes encoding major stress regulators, such as AtfA [58,64,65] and AslA [59], can be directly repressed by MsnA *in vivo*, at least under vegetative growth conditions, in the absence of stress stimuli (Fig 7E–7H). Taking the results together, it becomes clear that the role of MsnA is not solely restricted to the mediation and orchestration of salt-stress response, but also to the deactivation of corresponding signaling, by attenuating the expression of stress key regulators, when stress stimuli are no longer present. These results are in accordance with the findings of a former study, where was shown that several F-box proteins (receptors of substrates that are targeted by ubiquitin for degradation) required for stress are expressed while the fungus is growing vegetative without any stress stimulus [66]. Overall, this shows that the fungus just in anticipation of stress is able to produce proteins, such F-box or TFs, that are related with specific stress responses. Moreover, based on these findings, it would be interesting to examine the *in vivo* binding preferences of MsnA under salt-stress or other stress conditions in a genome-wide scale to see which genes are directly controlled by the TF.

**Fig 9:**
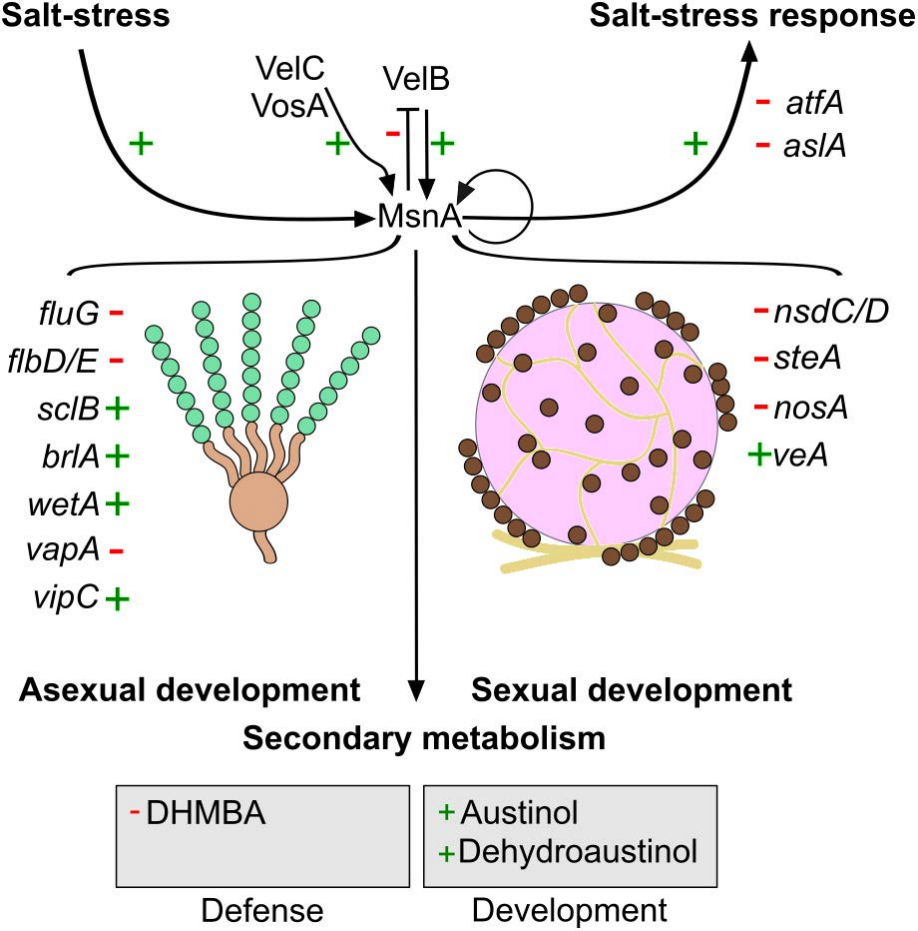
MsnA controls master regulators as well as regulatory gene networks to mediate developmental and salt-stress signals in *A. nidulans.* The illustrated model shows the global influence that MsnA has in different facets of *A. nidulans*` biology. Master regulators of the asexual (*fluG*, *flbs*, *sclB*, *blrA*, *wetA*, *vapA* and *VipC*) as well as sexual (*nsdC*/D, *steA*, *veA* and *nosA*) development, are directly (*in vivo*) transcriptionally controlled by MsnA. A dynamic, mutual cross-regulatory circuit between MsnA and VelB coordinates and mediates several signals into organized transcriptional responses. Although, stress responses (such as high concentration of NaCl) are solely mediated by MsnA without any implication of VelB. MsnA seems to keep the salt-stress signaling off, when no real stimulus is present, by the direct repression of crucial stress TFs such as AtfA and AslA. Secondary metabolism is also influenced by the function of MsnA. Transcriptional changes, provoked by MsnA eventually lead to changes in the final secondary metabolites (related to the defense or the development) of the corresponding metabolic pathways. Green crosses (+) depict the induction and red hyphens (-) the repression, caused by the direct *in vivo* association of MsnA to promoters of the corresponding genes. The construction of this cartoon figure was done by using the freely available vector graphics software of Inkscape (https://inkscape.org/).

We propose a model (Fig 9) where MsnA functions as an organizer of a transcriptional hub. Known prominent regulators of development and stress responses, such as the velvet proteins, but also other so far unknown regulators, can be influenced by MsnA or (and) can affect MsnA itself. Through these mutual transcriptional interactions at the MsnA regulatory hub, the orchestration of *A. nidulans* development, secondary metabolism and stress response can be coordinated based on signals that the fungus receives by its environment.

## Materials and methods

### Strains, media and growth conditions

The strains of *Aspergillus nidulans* and *Escherichia coli* used in this study are listed in the S1 and S2 Tables.

The *A. nidulans* strain used as a wildtype for all the experiments was AGB551. *A. nidulans* strains were cultivated in minimal medium composed of 1% (w/v) glucose, 2 mM MgSO_4_, 1x AspA (7 mM KCl, 70 mM NaNO_3_, 11.2 mM KH_2_PO_4_, pH 5.5), 0.1% (v/v) trace element solution (76 µM ZnSO_4_, 178 µM H_3_BO_4_, 25 µM MnCl_2_, 18 µM FeSO_4_, 7.1 µM CoCl_2_, 6.4 µM CuSO_4_, 6.2 µM Na_2_MoO_4_, 174 µM EDTA) and pH 5.5 [67]. Liquid medium was supplemented with 0.1% (v/v) pyridoxine and 5 mM uridine. For solid medium 2% agar and 0.1% (w/v) uracil was added. After transformation, *A. nidulans* protoplasts were plated on solid medium additionally containing 120 mg/mL nourseothricin as a selection marker. The selection-marker cassette was recycled from the *A*. *nidulans* genome after the selection process, by growing the strains on medium containing 0.5% (w/v) glucose and 0.5% (w/v) xylose.

The DH5α strain of *Escherichia coli* was used for cloning purposes of this study. The culture medium used for the cultivation of *E. coli* was a lysogeny broth (LB) [68] medium (1% tryptone, 0.5% yeast extract, 1% NaCl, supplemented with ampicillin (100 µg/mL as a final concentration) for the selection of transformants with the desired plasmid. In case of solid medium an addition of 2% (w/v) agar was used.

### Plasmid construction, handling and preparation

The extraction of the plasmid DNA was performed with the NucleoSpin Plasmid Kit (Macherey-Nagel, Düren, Germany), under the manufacturer`s instructions. The final plasmids, carrying the constructed cassettes were verified via Sanger sequencing by Microsynth Seqlab GmbH (Göttingen, Germany) and the use of the Lasergene software (DNA Star Inc., Madison, USA).

The backbone plasmid used for the construction of the cassettes, which were transformed into *A. nidulans* strains, was the pBluescript KS (pME4696), constructed and used initially by [69]. This plasmid carries a *Pml*I restriction site for the integration of the target genes 5`-flanking region and a *Swa*I site for the integration of its 3`-flanking region. The 5` and 3` flanking regions define the exact position in the *A. nidulans* genome, where the incorporation of the constructed cassette will take place via homologue recombination, after transformation into *A. nidulans*. For the amplification of the 5`and 3`flanking regions of all the cassettes used in this study, genomic DNA from the AGB551 wildtype strain was used as a template. To construct the final cassettes, both flanking regions were inserted into the pME4696 vector via the GeneArt Seamless Cloning and Assembly Kit (Invitrogen, Carlsbad, CA, USA), following the manufacturer`s instructions. The cassettes included a recyclable selection marker, which provided resistance to nourseothricin (natRM).

### Construction of the deletion cassette of *msnA* and the *A. nidulans* Δ*msnA* strain

The amplification of the 5’-flanking region was performed with the MB62/63 pair of primers, yielding a 1663 bp amplicon, spanning from just before an ATG triplet, encoding for the TSS (translation start site) of the *msnA* and extended upstream from that point. The amplification of the 3’-flanking region of the deletion cassette was performed with the MB64/65, which gave an amplicon of 1036 bp starting just after a TGA triplet encoding for the stop codon of *msnA* and extending downstream from that point. Next, the 5’ region and 3’ region flanking regions were cloned into the pME4696 empty vector, giving the final plasmid of pME5558. Final constructs carrying both of the flanking regions were verified by Sanger sequencing, prior to the excision of the cassette with the *Mss*I (*Pme*l) restriction enzyme from the pMB4-10 plasmid, that was used subsequently for transformation into the *A. nidulans* wildtype strain, resulting in the AGB1707 strain, after the selection marker was recycled.

### Construction of the complementation cassette *msnA:hinge:GFP:3*’*UTR*_*msnA*_*:trpC-terminator* and the *A. nidulans* Δ*msnA/*MsnA-GFP strain

The first fragment of this cassette is composed by several different parts amplified from different templates fused by fusion PCR based on fragment compatibility introduced during the primers design. In short, the initial amplicon-A including part of the promoter region of the *msnA* and the *msnA* gene was amplified using genomic DNA from AGB551 as a template and the primer pair of MB1029/1030, yielding a 5032 bp fragment (the triplet encoding for the stop codon of *msnA* was removed via primer designing). Next, using as a template plasmid DNA, derived from the vector pChS242 [70], the hinge-GFP fragment was amplified with the primers MB1031/1032 giving a 752 bp amplicon-B; hinge is a short (15 bp) spacer/non-coding part that simply distances the GFP from the protein that is going to be tagged with. At this point, amplicon-A and -B were fused by fusion PCR using the primer pair of MB1029/1032 yielding amplicon-C with the size of 5784 bp. Subsequently, an amplicon was generated, from genomic DNA of AGB551, with the primers MB1033/1034 in order to amplify specifically the 3`-UTR (as it was predicted in FungiDB database) of *msnA* producing amplicon-D. The amplification of a *trpC*-terminator by using plasmid DNA from pChS459 as a template and the primers MB1035/1036 followed, yielding a 1460 bp amplicon-E. In the next step the amplicons-D and -E were fused by using the primers MB1033/1036 giving a product of 1460 bp, amplicon-F. In the last step of the construction of the first part for this cassette, the amplicons -C and -F were fused by fusion PCR by using the primer of MB1029/1036 yielding a final fragment of 7244 bp. The 3`-flanking region of the gene, was amplified from genomic DNA with the primers MB1037/1038 and had a size of 2001 bp. The first fragment and the 3’ region flanking region were finally cloned into the pME4696 empty vector leading to the pME5559 vector. Final cassettes were verified ed by Sanger sequencing, prior its excision with the *Mss*I (*Pme*l) restriction enzyme. The derived cassette then was subsequently transformed into the *A. nidulans* AGB1707 (Δ*msnA*) strain, resulting in AGB1708, after the selection marker was recycled.

### Construction of the *A. nidulans* Δ*msnA* Δ*velB* double deletion strain

The cassette that was used for the construction of the Δ*msnA* (as described previously), was also used for the construction of the double deletion strain. The corresponding cassette was excised, with the *Mss*I restriction enzyme from the pME5558 plasmid. It was then transformed and integrated in locus into the genome of the Δ*velB* (AGB1064) strain (sharing the same genetic background with the AGB551 wild type the except the deletion of the *velB*). Positive clones from the corresponding transformation were selected, subsequently the selection marker was recycled and the strains checked genetically for carrying both deletions (Δ*msnA* and Δ*velB*) by Southern hybridization, leading finally to the AGB1709 *A. nidulans* strain.

### Phenotypical characterization

To examine the development of the fungus, 2000 for each strain were point inoculated in the middle of MM plates, supplemented with 0.1% (v/v) pyridoxine, 5 mM uridine, and 0.1% (w/v) uracil. After growth under asexual or sexual conditions for the indicated time, scans were made from the top and bottom of the plates.

For single cleistothecia images and closeup pictures of the colonies, the Axiolab microscope (Carl Zeiss Microscopy, Oberkochen, Germany) and the SZX12-ILLB2-200 binocular microscope (Olympus, Shinjuku, Japan) were used.

For quantification of conidia, the identical number of spores from all strains were distributed equally on solid medium. Cultures were incubated for the indicated time under asexual growth conditions. The spores from each strain were collected and measured with the Coulter Z2 particle counter (Beckman Coulter GmbH, Krefeld, Germany).

For quantification of cleistohecia, the identical number spores from all strains was distributed equally on plates supplemented with 0.1% (v/v) pyridoxine, 5 mM uridine, and 0.1% (w/v) uracil) MM. Cultures were grown for the indicated time under sexual growth conditions. Subsequently three agar plugs of 5 mm^2^ were removed from each plate (by using the larger side of 200 μL pipette tip) and placed on a new agar plate. The exact number of cleistothecia for each strain was then assessed by counting.

### ChIP, sequencing and data analysis

#### ChIP.

The ChIP experiments were performed with mycelia derived from submerged cultures. In the case of the ChIP-seq for MsnA, the Δ*msnA* MsnA-GFP complementation strain was used, expressing *msnA* fused with GFP (*Green Fluorescent Protein*) under its native promoter. Three different ChIP-seq experiment were performed with the Δ*msnA* MsnA-GFP strain, the 1^st^ under Vege, the 2^nd^ under Asex and the third under Sex growth conditions. In the case of the ChIP for VelB, the VelB-GFP strain was used, expressing *velB* fused with GFP under its native promoter. The Wt strain served as a negative control. For all the strains used for the ChIPs (Δ*msnA* MsnA-GFP, VelB-GFP and Wt), a total number of 5x10^8^ spores was initially inoculated in 500 mL liquid medium inside of 2 L flasks and grown for 20 hours under constant rotation, light and at 37 °C. For all four in total ChIP experiments, after the mycelia reached the indicated stage/time point, subsequently they were harvested quickly, dried and immersed in fixation solution containing 1% formaldehyde for 20 minutes. The whole ChIP experiments were performed as described in Sasse *et al.,* 2023 [70], with minor modifications for each ChIP, which are the following. For each ChIP-seq with MsnA, three independent biological replicates of Δ*msnA* MsnA-GFP were used. As inputs (negative controls) the samples after shearing of the chromatin without GFP antibody added were considered. For the ChIP with VelB, three independent biological replicates were used from the VelB-GFP strain and another three from the Wt strain (negative controls). The IPs of both strains were performed with GFP antibody. For the ChIPs with MsnA-GFP the input samples were subjected to the same DNA purification procedure as the samples derived from the IP with the GFP antibody, prior to library preparation for sequencing. For the VelB ChIP, a GFP antibody was applied to the IPs derived from VelB-GFP and Wt strains. All reagents, instruments, kits and rest of the procedures for both of the ChIP experiments were the same as in Sasse *et al*., 2023 [70].

#### Library preparation and NGS sequencing.

ChIP-seq libraries preparation and the following sequencing were performed at the NGS- Integrative Genomics Core Unit (NIG), University Medical Center Göttingen. Initially, the quantity and quality of ChIPed-DNA and input samples were determined by a Fragment Analyzer. The preparations of the ChIP-seq libraries were done by using the TruSeq ChIP Library Preparation Kit (Illumina, San Diego, USA), following manufactures` instructions. The size range of the final DNA libraries were assessed with the Fragment Analyzer, using the SS NGS Fragment 1-6000 bp Kit, with an average size of 340 bp. The Denovix system (Bio-Rad Laboratories, Hercules, CA, USA) was used for the quantification of DNA libraries. Libraries were amplified and sequenced on an S1 flow cell NovaSeq 6000 (Illumina, San Diego, USA), for 100 cycles. The sequencing images produced were processed with the BaseCaller Illumina software to generate BCL files. Those files were then demultiplexed into fastq files with bcl2fastq v2.20.0.422 generating a FastQC for data quality control.

The subsequent ChIP-seq analysis was performed partly by using the same software and pipelines as presented in Sasse *et al*., 2023 [70] with the minor modifications that are following. Part of the analysis was performed in the GALAXY platform [71] maintained by the GWDG (Gesellschaft für wissenschaftliche Datenverarbeitung mbH Göttingen). For the mapping of the raw sequences, derived from high-throughput sequencing, the *Aspergillus nidulans* genome (downloaded from fungidb.org: FungiDB-46_AnidulansFGSCA4_Genome.fasta) was used. Visualization of the ChIP-seq, in terms of mapped reads along the genome of the fungus, was performed with the use of the Integrative Genome Browser (IGB) [72]. The GO-enrichment analysis was performed with the ShinyGo v0.82 webtool [50]. The Venn diagrams were made by using the InteractiVenn web tool [73]. The raw sequencing data for the ChIP-seq experiment have been deposited at NCBI [BioProject ID PRJNA1260675].

### DNA extraction

The genomic DNA derived from *A. nidulans* mycelia grown overnight in liquid cultures under constant rotation, at 37 °C and in light. The extraction of genomic DNA was performed based to the protocol described by Thieme *et al*., 2018 [52]. All the concentrations of the purified DNAs were measured by NanoDrop ND-1000 spectrophotometer (Peqlab, Erlangen, Germany).

### Transformations *E. coli* and *A. nidulans*

Transformation of *E. coli* and *A. nidulans* strains were performed based on protocols described in Meister *et al*., 2019 [69]. Successful transformation of cassettes into the *A. nidulans* genome were confirmed by Southern hybridization as described by Southern, 1975 [74]. For Southern`s probe labelling the AlkPhos Direct Labelling Module (GE Healthcare Life Technologies, Little Chalfont, UK) was used following the manufacturer`s instruction.

### RNA extraction and cDNA synthesis

All strains were initially inoculated with the same number of spores (10^8^) in 100 mL liquid medium. Mycelia growing under Vege were dried in miracloth (Merck KGaA, Darmstadt, Germany). 100 mg were quickly weighted for each biological replicate and placed inside 2 mL reaction tubes with three zirconium oxide beads (SiLibeads; Sigmund Lindner GmbH, Warmensteinach, Germany). Samples were frozen instantly in liquid nitrogen. In case of RNA deriving from Asex and Sex conditions, the mycelia were transferred to solid MM after initial Vege growth. Cultures were incubated for the indicated time under asexual (light, 37 °C) or sexual (dark, 37 °C, parafilm around the plates) conditions. For each time point, mycelia were harvested and handled as described before. Sampled mycelia were grinded for 1 minute with a ball mill MM400 (Retsch, Haan, Germany), just before RNA extraction. The teflon cassettes-cases (2 mL epis holders) of the mill were precooled in -80°C for about an hour prior to placement of the frozen 2 mL tubes with the sampled mycelia for grinding. RNA extraction with the pulverized mycelia followed by using the RNeasy Plant Miniprep Kit (Qiagen) according to manufacturer`s instructions. The final concentration of the RNAs was assessed by using the NanoDrop ND-1000 spectrophotometer (Peqlab, Erlangen, Germany). The subsequent synthesis of cDNA was performed with 1.0 µg total RNA from each sample by using the QuantiTect Reverse Transcription Kit (Qiagen, Hilden, Germany) according to the manufacturer`s protocol.

### Quantitative real-time PCR

Quantitative real-time polymerase chain reaction (qRT-PCR) was employed for studying gene expression. The CFX Connect Real-Time System (Bio-Rad Laboratories, Hercules, CA, USA) was used. All cDNA templates were diluted 1:5 for the qRT-PCR, which was performed with the MESA GREEN qPCR MasterMix Plus for SYBR from EUROGENTEC (Lüttich, Belgium) by following the manufacturer`s instructions. Data was partially analyzed with the CFX Manager 3.1 software package from Bio-Rad Laboratories and partially with Excel (Microsoft, *Washington, USA*), using the ΔCt method with a reference gene 2Ct(reference)-Ct(target) (BioRad Laboratories, qRT Application Guide) method for relative quantification of gene expression. As reference gene, *h2A* (*histone2A*) was used. qRT-PCR experiments were conducted at least with a minimum of 3 biological replicates, each consisting of at least three technical replicates. All qRT-PCR primers, used in this study are listed in the S3 Table.

### Secondary metabolites analysis

A total number of 10^5^ spores was equally spread on solid medium. Colonies were grown for the indicated time under asexual and sexual growth conditions. For each strain, three plates were prepared (three replicates). The sampling, SM extraction, LC-MS and the subsequent analysis was performed as described by Liu *et al*, 2021 [47]. Specific details for all the all the detected secondary metabolites are listed in the S5 Table.

### Microscopy

For fluorescence microscopy, 3500 spores of each strain were inoculated in 400 µL liquid medium with supplements, placed in a single well of an 8-well chambered coverslip (Ibidi GmbH, Gräfelfing, Germany), incubated at 37 °C and light for 20 hours. Fluorescence microscopy was performed with the inverted confocal microscope Zeiss AxioObserver, Z.1 (Zeiss, Oberkochen, Germany), and the software SlideBook 6.0 software package (Intelligent Imaging Innovations GmbH, Göttingen, Germany). For staining of nuclei, the Hoechst dye (Invitrogen, Massachusetts, USA) was used.

### Figures processing

The processing of all figures was done by the vector-graphics editor Inkscape (Inkscape Project, 2020; Inkscape, available at https://inkscape.org).

## Supporting information

S1 Fig The MsnA regulator is a cytoplasmatic and a nuclear localized protein, which strongly affects growth`s colony formation during the development of *A. nidulans.* Phenotypical analysis of *A. nidulans* Δ*msnA* and complementation strain Δ*msnA* MsnA-GFP, under (A) 8-d asexual (constant light) and (B) 12-d sexual (dark) development promoting growth conditions. Scans in both cases derived from initial spot of 2000 conidia in the middle of the plate following incubation at 37 °C. Scale bars of photos at lower part of panel (B) of many cleistothecia show a size of 200 µm, the bars shown in images of single unbroken cleistothecia represent 100 µm length, and the ones included in the images of broken cleistothecia represent 50 µm length. Confocal live microscopy (C) of the complementation strain Δ*msnA* MsnA-GFP natively expressing functional MsnA-GFP; the scale bar represents length of 5 µm. Hyphae were grown Vege conditions. White arrow indicates the nuclear co-localization of the GFP signal with nuclear dye (Hoechst).
(TIF)

S2 Fig Proteins associated with the development, the cell wall organization and with several metabolic processes, are enriched among the top targets of MsnA, independent from the growth conditions. The dot plots (**A**-**C**) show the GO (Gene Ontology)-enrichment analysis, in terms of the biological process (BP). The analysis was performed with the webtool ShinyGO 0.82 (https://bioinformatics.sdstate.edu/go/), using as input for the common IDs of genes identified simultaneously in all three independent sets of the ChIP-seq analysis, for each of the ChIP-seqs performed under (**A**) Vege (860 IDs), (**B**) Asex (332 IDs) for and (**C**) Sex (744 IDs) growth.
(TIF)

S3 Fig MsnA influences directly, *in vivo*, the expression of genes encoding established *A. nidulans* master regulators in mycelia growing under asexual and sexual growth conditions. Screen shots from the Integrative Genome Browser (IGB) illustrating peaks from the ChIP-seq data with MsnA-GFP (**A**) growing under Asex and (**C**) Sex conditions, located into promoter regions of genes encoding for known key regulators of asexual and sexual development of the fungus. Black horizontal arrows, below the corresponding genes show the direction of their transcription each screen shot. Blue tracks represent the inputs (negative controls), green tracks the Asex and orange tracks the Sex growth correspondingly. Gene expression analyses, for the known regulators that found to be direct targets of MsnA, was performed via qRT-PCRs, with RNA derived from mycelia of wildtype (Wt) and Δ*msnA* strains grown either under (**B**) Asex or (**D**) Sex growth correspondingly. SclB, BrlA and WetA as also major regulators of asexual development are directly transcriptionally controlled by MsnA in mycelia derived Vege or Asex conditions as well. **(E)** Screen shots from the IGB showing ChIP-seq peaks from mycelia of MsnA-GFP growing either Vege (red tracks) or Asex growth (green tracks). Gene expression analysis for the genes *sclB*, *brlA* (both of its functional overlapping transcripts, *brlAα* and *brlAβ*) and *wetA* (**F** and **G**) performed by qRT-PCRs. RNAs derived from wildtype (Wt) and Δ*msnA* strains, growing either under Vege (**F**) or Asex (**G**) growth conditions. Each qRT-PCR presented in this figure, represents at least three biological replicates per strain and per different time point. Each biological replicate consists of minimum three technical replicates. Statistical differences for the gene expression data were performed by t-test, with *: p < 0.05, **: p < 0.01 and ***: p < 0.001.
(TIF)

S4 Fig MsnA is associated with the same DNA motif across all different growth conditions, vegetative, asexual and sexual. Logos that are presented in this figure are showing the top ranked *de novo* motifs, as discovered by employing the RSAT-peak-motif tool. *De novo* motif discovery was performed for all three independent sets of each of the ChIP-seq performed in the three different growth conditions of Vege, Asex and Sex growth. For each independent set a group of the 100 bp sequences was used as input of the RSAT tool. Each of these sequences were located underneath the summit of the top 150 ChIP-seq peaks. All of those peaks were located into 3 kb promoter regions. Here are presented the rest two top de novo discovered motifs from the remaining two sets of analysis for each ChIP-seq that are not presented in the panel **D** of Fig 2.
(TIF)

S1 Table *A. nidulans* strains used in this study. (XLSX)

S2 Table *E. coli* used in this study. (XLSX)

S3 Table Primers used in this study. (XLSX)

S4 Table ChIP-seq common locus IDs with peaks up to 3 kb promoter regions. (XLSX)

S5 Table Secondary metabolites controlled by MsnA under asexual growth conditions, as detected by detected by LC-MS/MS. (XLSX)
