# Supplementary figures and images for: Molecular circuit between Aspergillus nidulans transcription factors MsnA and VelB to coordinate fungal stress and developmental responses
Source: PLoS Genet. 2025 Jul 17;21(7):e1011578. doi: 10.1371/journal.pgen.1011578 (PMC12270170; doi:10.1371/journal.pgen.1011578)

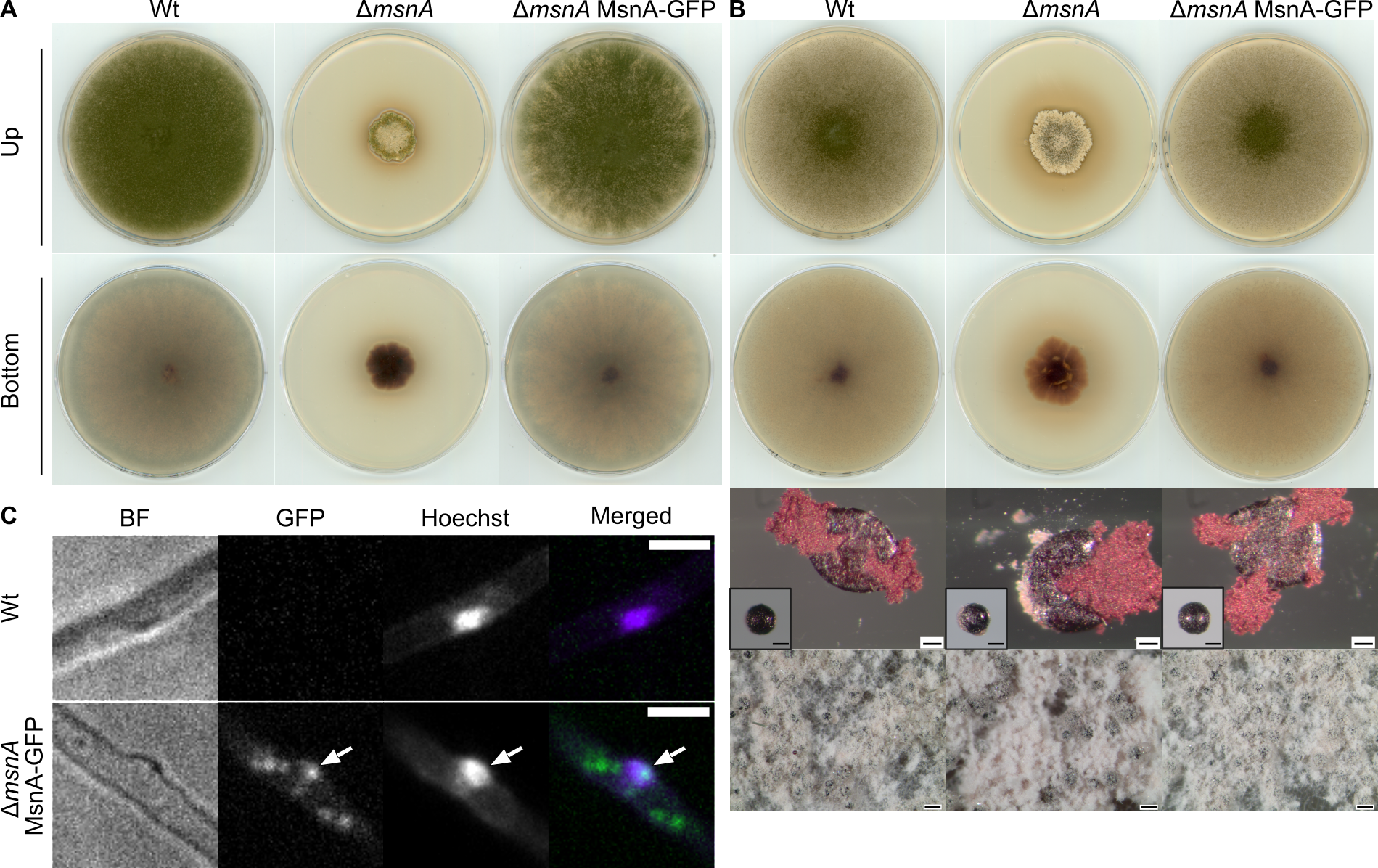

Supplement: S1 Fig — Phenotypical analysis of A. nidulans ΔmsnA and complementation strain ΔmsnA MsnA-GFP, under (A) 8-d asexual (constant light) and (B) 12-d sexual (dark) development promoting growth conditions. Scans in both cases derived from initial spot of 2000 conidia in the middle of the plate following incubation at 37 °C. Scale bars of photos at lower part of panel (B) of many cleistothecia show a size of 200 µm, the bars shown in images of single unbroken cleistothecia represent 100 µm length, and the ones included in the images of broken cleistothecia represent 50 µm length. Confocal live microscopy (C) of the complementation strain ΔmsnA MsnA-GFP natively expressing functional MsnA-GFP; the scale bar represents length of 5 µm. Hyphae were grown Vege conditions. White arrow indicates the nuclear co-localization of the GFP signal with nuclear dye (Hoechst). (TIF) [file pgen.1011578.s001.tif]

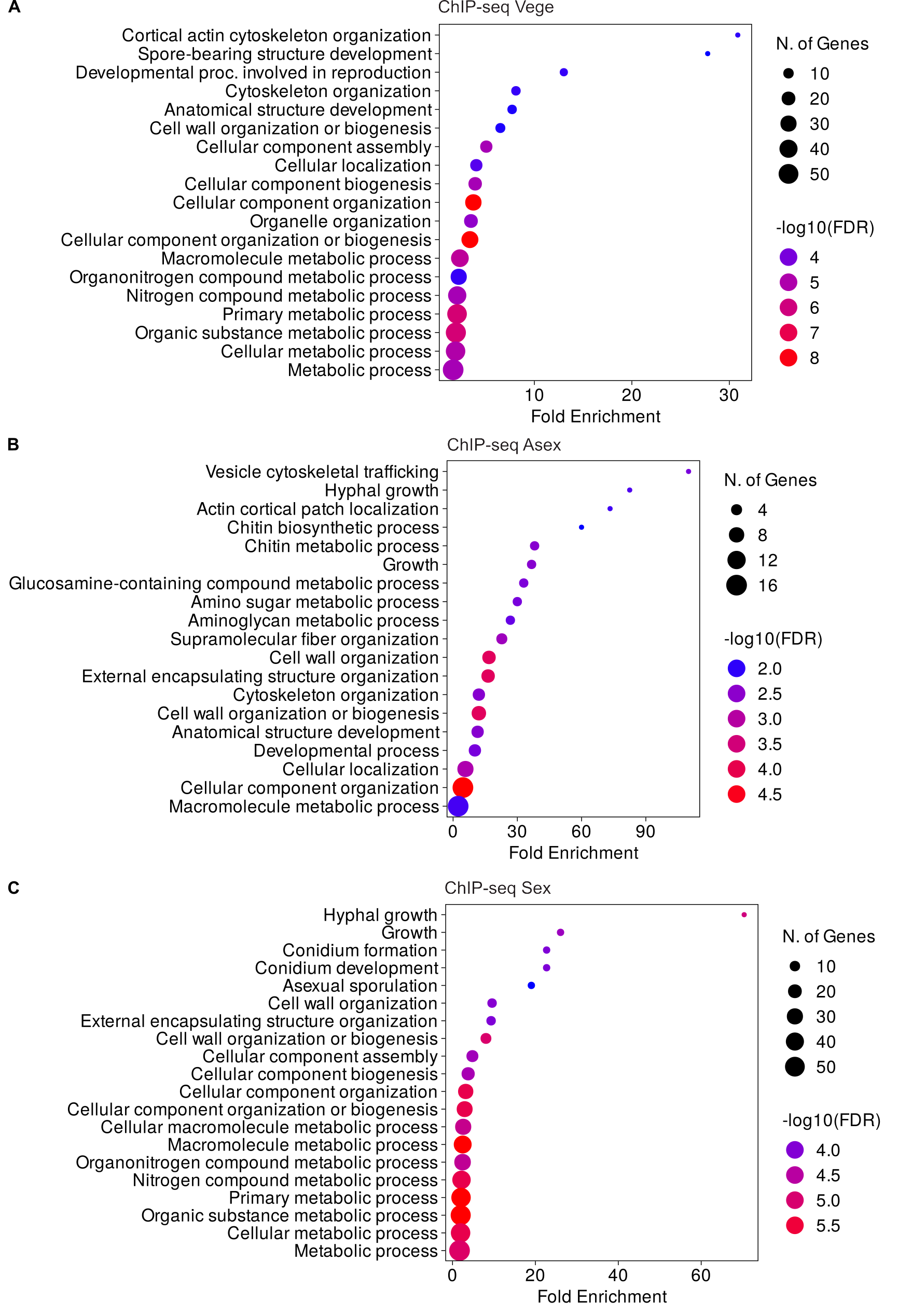

Supplement: S2 Fig — The dot plots (A-C) show the GO (Gene Ontology)-enrichment analysis, in terms of the biological process (BP). The analysis was performed with the webtool ShinyGO 0.82 (https://bioinformatics.sdstate.edu/go/), using as input for the common IDs of genes identified simultaneously in all three independent sets of the ChIP-seq analysis, for each of the ChIP-seqs performed under (A) Vege (860 IDs), (B) Asex (332 IDs) for and (C) Sex (744 IDs) growth. (TIF) [file pgen.1011578.s002.tif]

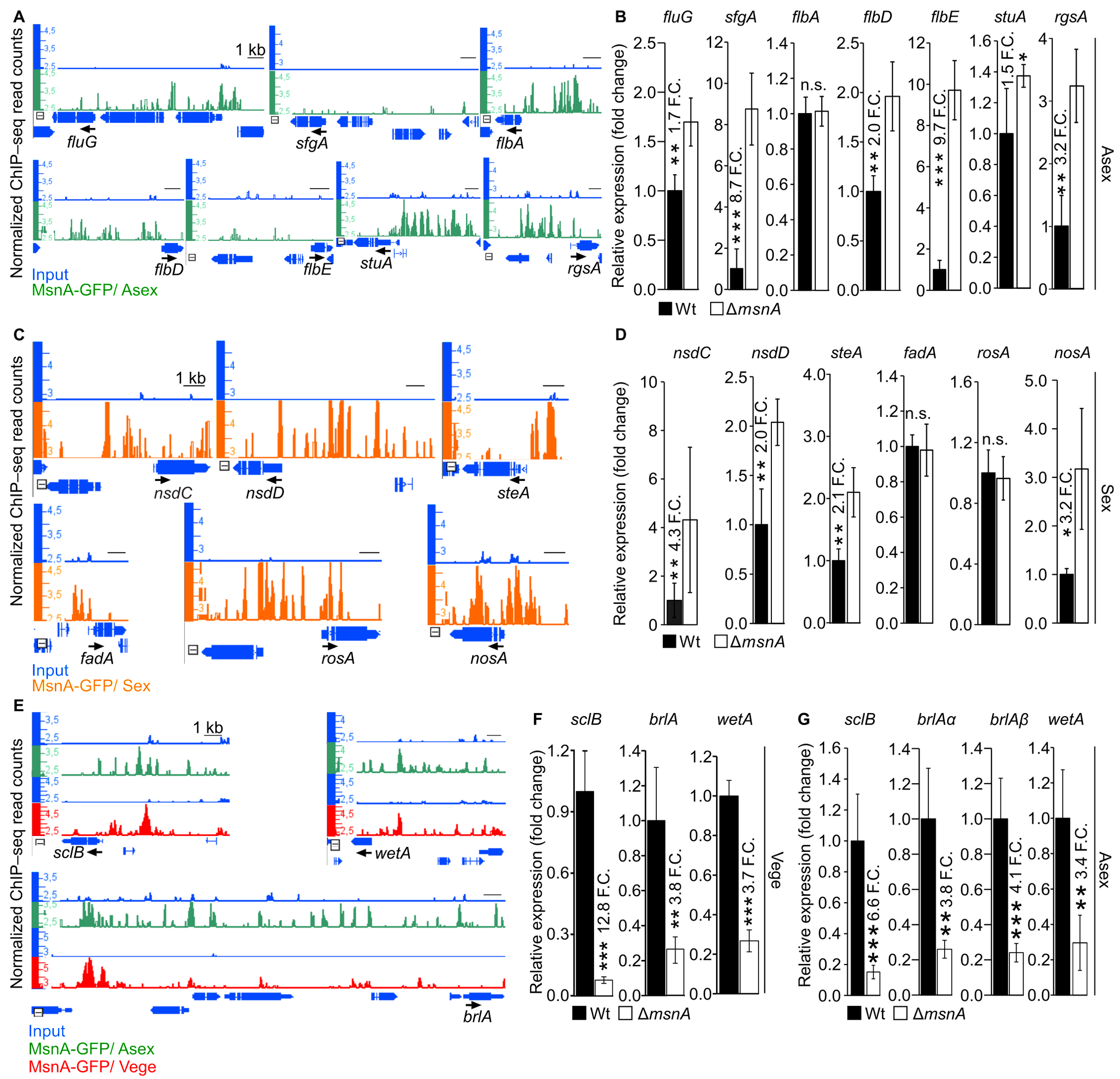

Supplement: S3 Fig — Screen shots from the Integrative Genome Browser (IGB) illustrating peaks from the ChIP-seq data with MsnA-GFP (A) growing under Asex and (C) Sex conditions, located into promoter regions of genes encoding for known key regulators of asexual and sexual development of the fungus. Black horizontal arrows, below the corresponding genes show the direction of their transcription each screen shot. Blue tracks represent the inputs (negative controls), green tracks the Asex and orange tracks the Sex growth correspondingly. Gene expression analyses, for the known regulators that found to be direct targets of MsnA, was performed via qRT-PCRs, with RNA derived from mycelia of wildtype (Wt) and ΔmsnA strains grown either under (B) Asex or (D) Sex growth correspondingly. SclB, BrlA and WetA as also major regulators of asexual development are directly transcriptionally controlled by MsnA in mycelia derived Vege or Asex conditions as well. (E) Screen shots from the IGB showing ChIP-seq peaks from mycelia of MsnA-GFP growing either Vege (red tracks) or Asex growth (green tracks). Gene expression analysis for the genes sclB, brlA (both of its functional overlapping transcripts, brlAα and brlAβ) and wetA (F and G) performed by qRT-PCRs. RNAs derived from wildtype (Wt) and ΔmsnA strains, growing either under Vege (F) or Asex (G) growth conditions. Each qRT-PCR presented in this figure, represents at least three biological replicates per strain and per different time point. Each biological replicate consists of minimum three technical replicates. Statistical differences for the gene expression data were performed by t-test, with *: p < 0.05, **: p < 0.01 and ***: p < 0.001. (TIF) [file pgen.1011578.s003.tif]

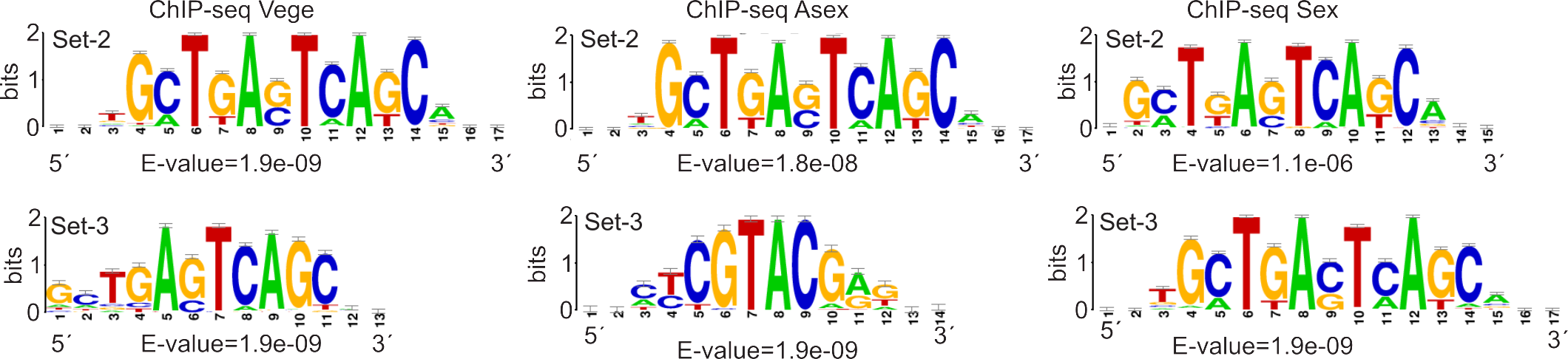

Supplement: S4 Fig — Logos that are presented in this figure are showing the top ranked de novo motifs, as discovered by employing the RSAT-peak-motif tool. De novo motif discovery was performed for all three independent sets of each of the ChIP-seq performed in the three different growth conditions of Vege, Asex and Sex growth. For each independent set a group of the 100 bp sequences was used as input of the RSAT tool. Each of these sequences were located underneath the summit of the top 150 ChIP-seq peaks. All of those peaks were located into 3 kb promoter regions. Here are presented the rest two top de novo discovered motifs from the remaining two sets of analysis for each ChIP-seq that are not presented in the panel D of Fig 2. (TIF) [file pgen.1011578.s004.tif]
